# Dual Emissive Ir(III) Complexes for Photodynamic Therapy and Bioimaging

**DOI:** 10.3390/pharmaceutics13091382

**Published:** 2021-09-01

**Authors:** Marta Redrado, Andrea Benedi, Isabel Marzo, M. Concepción Gimeno, Vanesa Fernández-Moreira

**Affiliations:** 1 Departamento de Química Inorgánica, Instituto de Síntesis Química y Catálisis Homogénea (ISQCH), CSIC-Universidad de Zaragoza, 50009 Zaragoza, Spain; marta@unizar.es; 2 Departamento de Bioquímica y Biología Celular, Universidad de Zaragoza-CSIC, 50009 Zaragoza, Spain; andreabenedi94@gmail.com (A.B.); imarzo@unizar.es (I.M.)

**Keywords:** photodynamic therapy, iridium, fluorescence microscopy, mitochondria, cytotoxicity, optical properties, dual emiter

## Abstract

Photodynamic therapy (PDT) is a cancer treatment still bearing enormous prospects of improvement. Within the toolbox of PDT, developing photosensitizers (PSs) that can specifically reach tumor cells and promote the generation of high concentration of reactive oxygen species (ROS) is a constant research goal. Mitochondria is known as a highly appealing target for PSs, thus being able to assess the biodistribution of the PSs prior to its light activation would be crucial for therapeutic maximization. Bifunctional Ir(III) complexes of the type [Ir(C^N)_2_(N^N-R)]^+^, where N^C is either phenylpyridine (ppy) or benzoquinoline (bzq), N^N is 2,2′-dipyridylamine (dpa) and R either anthracene (**1** and **3**) or acridine (**2** and **4**), have been developed as novel trackable PSs agents. Activation of the tracking or therapeutic function could be achieved specifically by irradiating the complex with a different light wavelength (405 nm vs. 470 nm respectively). Only complex **4** ([Ir(bzq)_2_(dpa-acr)]^+^) clearly showed dual emissive pattern, acridine based emission between 407–450 nm vs. Ir(III) based emission between 521 and 547 nm. The sensitivity of A549 lung cancer cells to **4** evidenced the importance of involving the metal center within the activation process of the PS, reaching values of photosensitivity over 110 times higher than in dark conditions. Moreover, complex **4** promoted apoptotic cell death and possibly the paraptotic pathway, as well as higher ROS generation under irradiation than in dark conditions. Complexes **2**–**4** accumulated in the mitochondria but species **2** and **4** also localizes in other subcellular organelles.

## 1. Introduction

**Figure 1:**
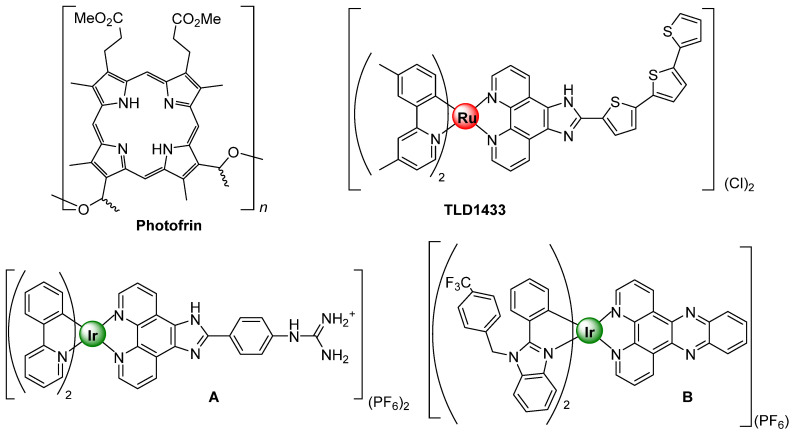
Chemical structures of Photofrin, TLD1433, and complexes (**A**,**B**) adapted from [8,18,22,23].

Photodynamic therapy is an underdeveloped cancer treatment that is attracting much attention recently due to the great prospect for destroying tumors and tumor vasculature as well as stimulating the immune response [1,2,3]. This treatment modality is based in three key pillars, a photosensitizer (PS), light and oxygen, rendering toxicity limited to the regions where the three components are together. Traditionally, organic scaffolds such porphyrins, chlorin or bacteriochlorin derivatives are used as PSs relying on their efficiency to generate highly toxic reactive oxygen species (ROS) upon irradiation at a specific light wavelength in presence of molecular oxygen [4]. Ideally, an optimum PSs should compile some common features like: (a) have strong absorption in the red/near infrared (NIR) region of the electromagnetic spectrum to allow the treatment of inner and bigger tumors; (b) have high quantum yield of the triple excited state formation and relatively long lifetime to generate ROS species effectively; (c) have minimum dark toxicity as well as a fast clearance from the body in order to avoid additional side effect to the patients and, last but not less important, (d) have a short and highly yielding synthetic route affording single and well characterized PSs [5]. Lately, transition metal complexes based on Ru, Os and Ir, among others, have been investigated as alternative to commercially available organic PSs to fulfill these premises [6]. In general, transition d^6^ metal complexes have tunable photophysical properties: high kinetic stability and low photobleaching character. The presence of the metal helps spin–orbit coupling, leading to ultrafast and efficient population of triplet excited states, and promoting high yields of singlet oxygen generation [7]. The successful incorporation in clinical trials of a Ru(II) complex, specifically TLD1433, developed by Prof. McFarland and coworkers for the treatment of bladder cancer with PDT, has encouraged to further investigate in this field, Figure 1 [8]. In addition to Ru(II) species, also Ir(III) complexes have demonstrated their great capacity as PSs [9]. A throughout design leads to Ir(III) complexes exhibiting high quantum yields for the triplet excited state and thus enabling the efficient generation of ROS species [10]. Alternatively, to this therapy function, Ir(III) complexes have also demonstrated to be suitable luminescent probes to target different organelles within the cells. Thus, many of them have been described to selectively localize preferentially in mitochondria [11], nuclei [12] or lysosomes [13] among other inner compartments, or even pass through one organelle to another [14] by simple modification of their ligand scaffolds.

In addition to that, many reports suggest that a PS targeting mitochondria is prone to enhance their potency of photodynamic therapy due to the distinct biological features of mitochondria [15]. In fact, there is a current trend in drug design for the development of mitochondrial targeted drugs due to their indispensable role in the regulation of cell functions [16]. Mitochondria are decisive regulators of apoptosis, and they produce most of the cell’s energy. They contain a high concentration of oxygen, which is one of the three main pillars of PDT. It was demonstrated that even low levels of singlet oxygen produced in the mitochondria are more toxic than large amounts produced in other parts of the cells like cell membrane or nucleus [17]. Actually, many of the commercially available PSs, such as Photofrin [18], Verteporfin [19] or Redaporfin [20,21], partially target mitochondria, where they display the therapeutic potential (Figure 1). In this sense being able to assess the biodistribution of the probe to ensure its successful delivery to mitochondria prior activating the therapeutic function will be key for maximizing its therapeutic potential.

**Figure 2:**
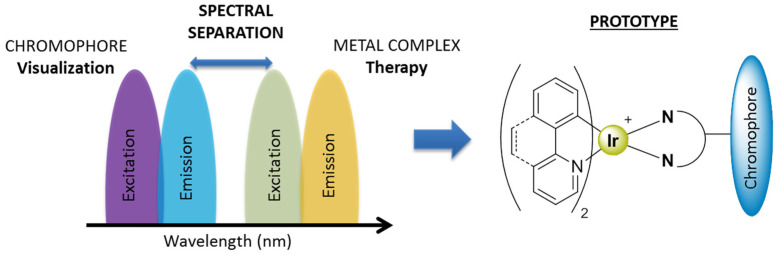
Depiction of the devised dual emissive Ir(III) PSs.

With all this knowledge in mind, we propose to develop a bifunctional mitochondrial selective Ir(III) complex that can act as both, PSs and cell imaging probe, by activating each function with a different irradiation wavelength. The design approach relies on the incorporation of a luminescent tag to an Ir(III) PSs with well differentiated optical properties from that of the metallic fragment, i.e., a spectral photophysical separation should be taken into consideration (Figure 2). For doing so, an organic chromophore would be coupled to any ligand within the Ir(III) coordination sphere. Typically, Ir(III) complexes for PSs rely on the structure of cationic species derived from [Ir(C^N)_2_(N^N)]^+^, where C^N represents an orthometallated and N^N a bisimine ligand. Therefore, taking the synthetic versatility that offers the bisimine 2,2′-dipyridylamine (dpa), where a chromophore like anthracene could be easily coupled to the amine as previously described by Zhua [24], we envisioned that it would be feasible the incorporation of analogous chromophores (acridine) using a similar methodology. In this way, an organic chromophore could be incorporated to the Ir(III) metallic scaffold offering the possibility of tracking the complex within the cells, using a different irradiation light wavelength from that of therapy activation. This work is aimed at delivering the first synthetic steps towards a rationale design of bifunctional metalloprobes within the area o PTD and cell imaging agents.

## 2. Materials and Methods

### 2.1. Instrumentation

^1^H and ^13^C-{^1^H} NMR, including 2D experiments, were recorded at room temperature (rt) on a BRUKER AVANCE 300 spectrometer (Bruker, Billerica, MA, USA) (^1^H-300 MHz, ^13^C-75 MHz) with chemical shifts (δ, ppm) reported relative to the solvent peaks of the deuterated solvent [25]. Mass spectra were recorded on a BRUKER ESQUIRE 3000 PLUS (Bruker, Boston, MA, USA), with the electrospray (ESI) technique. Steady-state photoluminescence analysis was performed in a Jobin-Yvon-Horiba fluorolog FL-3-11 spectrometer (Jobin Ybon Inc., Edison, NJ, USA). UV−Vis data were collected on an Evolution 600 spectrophotometer (Thermo Electron Scientifc Instrument LLC., Madison, WI, USA) using 1 cm quartz cells. Quantum yields were measured in a Hamamatsu Photonics Quantaurus-QY C11347-11 (Hamamatsu Photonics K.K., Hamamatsu City, Japan) via an absolute method, using an excitation scanning mode. The specific procedure entailed the measurement of each sample in aerated DMSO solution at room temperature (rt) after recording the reference sample (neat DSMO) in the same conditions. This procedure was repeated three times to confirm reproducibility and the quantum yield value given was this obtained at 440 nm excitation.

### 2.2. Cell Culture

Human lung carcinoma A549 cell line (from ATCC, Manassas, VA, USA) was cultured in high glucose Dubecco’s Modified Eagle’s Medium (DMEM) supplemented with 5% fetal bovine serum (FBS) as well as l-glutamine and penicillin/streptomycin at 37 °C in a humidified atmosphere of 95% air and 5% CO_2_.

### 2.3. Cell Viability Assays

Cell metabolic activity was analyzed by an MTT-reduction assay to assess the A549 cell sensitivity to the complexes. Thus, 10^5^ cells(A549)/mL were seeded in flat-bottom 96-well plates (100 µL/well) in complete medium and allowed to attach for 24 h. A stock solution of the complexes was prepared in DMSO 0.1 M. Subsequent dilutions of the different stock solutions from the complexes using DMEM were prepared. 100 µL/well were added to the cells, in concentrations ranging from 0.2 to 50 µM. each concentration was performed by quadruplicate. After 5 h of incubation with the complexes, duplicates of each concentration were irradiated with an LED lamp upon 405 and 470 nm with a light dose of 6.5 and 15.1 J/m^2^, respectively, for 10 min. Before irradiation, culture medium was replaced with fresh medium in order to avoid interference with non-internalized complexes. Cells were cultured for a further 19 h. 10 µL of MTT (5 mg ml**^−^**^1^ in PBS) were added to each well and then further 2 h incubation was allowed at 37 °C. Finally, after removing the culture medium, DMSO (100 µL/well) was added to dissolve the formazan crystals. Multiskan EX 96-well multiscanner autoreader (Thermo Fisher Scientific, Waltham, MA, USA) was used to determine the optical density at 550 nm and IC_50_ was calculated. Each experiment was repeated at least three independent analyses.

### 2.4. Cytotoxicity Assays

Necrotic and apoptotic cell death was determined by measuring cell membrane permeabilization and phosphatidylserine exposure on cell surface in A549 cells, respectively. 10^5^ cells/well were seeded in flat-bottom, 12-well plates (1 mL/well) in complete medium and left 12 h to attach to the plates. Cells were incubated with complex 4 for 5 h, at concentrations of the IC_50_, ½ IC_50_ and ¼ IC_50_ in quadruplicate before changing culture medium and irradiating at 470 nm of half of the experimental points. After 19 h, cells were trypsinized and thereafter resuspended in 50 μL of a mixture of DY634-conjugated Annexin V, Annexin-binding buffer (ABB; 140 mM NaCl, 2.5 mM CaCl_2_, 10 mM HEPES/NaOH pH 7.4) and 7-amino-actinomycin D (7-AAD). After incubation for further 15 min at rt in dark, cells were diluted to 300 μL with ABB. A total of 10,000 cells were acquired on a FACSCalibur^TM^ flow cytometer (BD Biosciences, San Jose, CA, USA) and cell death analysis was performed using Flowjo 7.6.1 (Becton Dickinson (BD), Franklin Lakes, NJ, USA), CellQuest Pro (BD Biosciences) and GraphPad Prism 8 (GraphPad Software, San Diego, CA, USA) softwares.

### 2.5. ROS Production Measurement

A549 cells (4 × 10^5^ cells/well) were seeded in flat-bottom, 6-well plates (3 mL/well) in complete medium and allowed attaching for 24 h. thereafter a DMSO solution of complex **4** added to cells up to concentration of IC_50_, ½ IC_50_ and ¼ IC_50_ in duplicate. Cells were incubated with the complex for 5 h and subsequently half of the experimental points were irradiated at 470 nm after replacing culture medium. Upon 19 h, cells were trypsinized, resuspended in 100 μL of a mixture of PBS and 2 μM of the dihydroethidium (DHE) probe, incubated at 37 °C in the dark for 15 min and diluted to 300 μL with PBS. Lastly, a total of 10,000 events were acquired on a FACSCalibur^TM^ flow cytometer and data were analyzed using the aforementioned softwares

### 2.6. Cell Cycle Analysis

4 × 105. A549 cells/well were seeded in complete medium in flat-bottom, 6-well plates (3 mL/well), left overnight to be attached to the bottom and incubated with complex **4** at the concentration of ¼ IC_50_ for 5 h in quadruplicate. Thereupon, cell culture was changed, and half of the experimental points were irradiated at 470 nm. After 19 h in culture, cells were trypsinized, washed twice with PBS, fixed with cold 70% ethanol (*v*/*v*) by vortexing and stored at −20 °C for at least 24 h. After that, cells were wash with cold PBS and stained with 500 μL of a solution of propidium iodide (PI) and RNase (Immunostep, Salamanca, Spain) for 20 min at rt in the dark. Finally, a total of 10,000 cells were acquired on a FACSCalibur^TM^ flow cytometer and data were analyzed using the aforementioned softwares.

### 2.7. Fluorescence Confocal Microscopy

A549 cells (8 × 10^3^ cells/well) were seeded in µ-slide 8 well (ibiTreat) (300 µL/well) in complete medium and left 24 h to get attached to the bottom of the slide. After removing 200 µL of culture medium from each well, 100 µL of a solution of the corresponding complexes were added reaching a final concentration of 2 µM. The complexes were incubated with the cells for 24 h, and thereafter, the internal standard MitoTracker Red (MTR) was added to a final concentration of 15 nM. MTR was incubated with the cells for further 15 min at rt. Finally, the medium of each well was replaced with fresh medium phenol red free. Images were collected in a ZEISS LSM 880 confocal microscope in a sequential mode with a 40 oil immersion lens. Specifically, it was used a line average of 4, a format of 1024 × 1024 pixels and a excitation wavelength of either 458 nm or 588 nm with a confocal pinhole of 1 Airy unit. Images were analyzed ZEISS ZEN lite (blue edition) a free software.

### 2.8. Distribution Coefficient (logP_7.4_)

The n-octanol-water coefficient of complexes **1**–**4** and **L1** and **L2** were determined using a shake-flask method [26]. A mixture of buffered-saline distilled water (100 mL, phosphate buffer [PO_4_^3−^] = 10 mM, [NaCl] = 0.15 M, pH 7.4) and n-octanol (100 mL) was shaken for 72 h to allow saturation of both phases. Approximately 0.5 mg of the complexes or ligands werw dissolved in 5 mL of the aqueous phase and 5 mL of the organic phase were added, mixing for 10 min. The resulting emulsion was centrifuged to separate the phases. The concentration of the compounds in each phase was determined using UV absorbance spectroscopy at 298 K. logP_7.4_ was defined as log{[compound(organic phase)]/[compound(aqueous phase)]}.

### 2.9. Starting Materials

The starting material [Ir(ppy)_2_(µ-Cl)]_2_ and [Ir(bzq)_2_(µ-Cl)]_2_ [27], were prepared according to published procedures. Each experimental data agrees with that already reported somewhere else. Other reagents and solvents were commercially available from Sigma-Aldrich, and used without further purification. Manipulations were performed under argon atmosphere.

### 2.10. General Synthetic Procedures

#### 2.10.1. Synthetic Procedure of Ligands **L1** and **L2**

An equimolar mixture of 2,2′-dipyridylamine (100 mg; 0.584 mmol) and 9-bromoanthracene (L1) (151.7 mg; 0.584 mmol) or 9-chloroacridine (L2) (128.52 mg; 0.584 mmol) was solved in 5 ml of toluene, when it became soluble, potassium *tert*-butoxide (77 mg; 0.65 mmol) was added to the solution. The reaction mixture was refluxed overnight. The volume of toluene was reduced under vacuum, affording a pale solid, which was filtered and dried affording L1 (162.2 mg; 80% yield) or L2 (196.76 mg; 96% yield). L1: ^1^H NMR (300 MHz, Acetone-d_6_) δ 8.69 (s, 1H), 8.50 (d, *J* = 8.9 Hz, 2H), 8.22 (d, *J* = 3.9 Hz, 2H), 8.16 (d, *J* = 8.4 Hz, 2H), 7.82 (d, *J* = 8.4 Hz, 2H), 7.74–7.56 (m, 6H), 6.89–6.80 (m, 2H) ppm. ^13^C NMR (75 MHz, Acetone-d_6_) δ 149.6, 148.40, 140.8, 138.2, 137.5, 129.8, 128.6, 128.5, 127.9, 126.9, 126.8, 116.8, 112.6, 103.0 ppm. L2: ^1^H NMR (300 MHz, Acetone-d_6_) δ 8.48 (d, *J* = 8.8 Hz, 2H), 8.25–8.20 (m, 4H), 7.96–7.90 (m, 2H), 7.84–7.75 (m, 4H), 7.68–7.61 (m, 2H), 6.85 (ddd, *J* = 7.2, 5.0, 1.0 Hz, 2H) ppm. ^13^C NMR (75 MHz, Acetone-d_6_) δ 148.4, 138.2, 131.6, 130.9, 128.4, 125.1, 116.8, 112.6, 110.4, 110.4 ppm. HRMS (ESI): *m*/*z* calcd. for C_23_H_16_N_4_ 348.1375; found: 349.1453 (L2 + H).

#### 2.10.2. Synthetic Procedure of Complexes **1**–**2**

[Ir(ppy)_2_(µ-Cl)]_2_ (50 mg, 0.046 mmol) was solved in 5 mL of MeOH, then L1 or L2 was added to the solution in an equimolar proportion (L1, 32.38 mg or L2, 32.47 mg). The reaction mixture was refluxed for 15 h in argon atmosphere. The resultant suspension was cooled, and the yellow solid was isolated by filtration affording 40.4 mg of 1 (92% yield) or 41.19 mg of 2 (94% yield) respectively. Complex 1: ^1^H NMR (300 MHz, CD_2_Cl_2_) δ 8.43 (dd, *J* = 9.1, 0.9 Hz, 3H), 8.13 (ddd, *J* = 5.8, 1.6, 0.8 Hz, 2H), 7.98–7.94 (m, 2H), 7.85 (ddd, *J* = 8.4, 1.5, 0.8 Hz, 2H), 7.73 (ddd, *J* = 8.2, 7.3, 1.5 Hz, 2H), 7.60–7.39 (m, 12H), 7.04 (ddd, *J* = 7.4, 5.8, 1.5 Hz, 2H), 6.89 (ddd, *J* = 7.8, 7.3, 1.3 Hz, 2H), 6.74 (td, *J* = 7.4, 1.4 Hz, 2H), 6.42 (t, *J* = 6.5 Hz, 2H), 6.08 (ddd, *J* = 7.5, 1.3, 0.5 Hz, 2H) ppm. ^13^C NMR (75 MHz, CD_2_Cl_2_) δ 168.3, 168.3, 163.0, 152.9, 150.0, 150.0, 149.8, 149.8, 144.5, 144.5, 138.8, 138.8, 138.5, 138.5, 132.3, 132.3, 130.8, 130.8, 129.2, 129.2, 127.9, 127.9, 127.8, 127.8, 127.77, 127.7, 126.3, 126.3, 125.2, 125.2, 123.4, 123.4, 122.7, 122.7, 120.2, 120.2, 117.9 ppm. Complex 2: ^1^H NMR (300 MHz, CD_2_Cl_2_) δ 8.46–8.30 (m, 6H), 8.21 (d, *J* = 8.2 Hz, 2H), 8.00 (t, *J* = 7.7 Hz, 2H), 7.84–7.52 (m, 10H), 7.33 (dd, *J* = 7.4, 5.8 Hz, 2H), 7.25 (dd, *J* = 8.2, 6.8 Hz, 2H), 6.94 (t, *J* = 7.5 Hz, 2H), 6.81 (t, *J* = 7.4 Hz, 2H), 6.69 (t, *J* = 6.5 Hz, 2H), 6.20 (d, *J* = 7.6 Hz, 2H) ppm. ^13^C NMR (75 MHz, CD_2_Cl_2_) δ 167.7, 152.1, 152.0, 150.4, 149.4, 149.3, 149.2, 143.9, 138.5, 138.0, 133.4, 131.7, 130.3, 126.5, 124.7, 122.9, 122.2, 121.5, 119.7, 118.4, 117.3, 116.9 ppm. HRMS (ESI): *m*/*z* calcd. for C_45_H_32_IrN_6_ 849.2318; found: 872.5459 (2 + Na).

#### 2.10.3. Synthetic Procedure of Complexes **3**–**4**

[Ir(bzq)_2_(µ-Cl)]_2_ (50 mg, 0.043 mmol) was solved in 5 mL of MeOH, then L1 or L2 was added to the solution in an equimolar proportion (L1, 29.72 mg or L2, 29.94 mg). The reaction mixture was refluxed for 15 h in argon atmosphere. The resultant suspension was cooled, and the yellow solid was isolated by filtration affording 24.6 mg of 3 (61% yield) or 40.08 mg of 4 (99% yield) respectively. Complex 3: ^1^H NMR (300 MHz, CD_2_Cl_2_) δ 8.59–8.47 (m, 5H), 8.38–8.31 (m, 2H), 8.25 (d, *J* = 8.6 Hz, 2H), 8.04 (dd, *J* = 8.4, 1.3 Hz, 2H), 7.83 (d, *J* = 8.8 Hz, 2H), 7.72 (d, *J* = 8.8 Hz, 2H), 7.67–7.48 (m, 8H), 7.41 (dd, *J* = 8.0, 0.9 Hz, 2H), 7.03 (dd, *J* = 7.9, 7.2 Hz, 2H), 6.45–6.39 (m, 2H), 6.08 (dd, *J* = 7.2, 0.9 Hz, 2H) ppm. ^13^C NMR (75 MHz, CD_2_Cl_2_) δ 171.9, 149.7, 148.7, 146.9, 145.9, 140.7, 138.5, 137.0, 129.7, 129.4, 129.0, 128.6, 127.4, 127.3, 127.2, 125.7, 123.8, 121.9, 120.2, 118.3, 117.0, 111.8, 105.7 ppm. Complex 4: ^1^H NMR (300 MHz, CD_2_Cl_2_) δ 8.54 (d, *J* = 5.3 Hz, 2H), 8.47 (t, *J* = 9.5 Hz, 4H), 8.33 (d, *J* = 8.1 Hz, 2H), 8.25 (d, *J* = 1.2 Hz, 2H), 7.86–7.82 (m, 4H), 7.71–7.68 (m, 4H), 7.60–7.49 (m, 6H), 7.39 (d, *J* = 7.8 Hz, 2H), 7.04 (t, *J* = 7.5 Hz, 2H), 6.46–6.38 (m, 2H), 6.08 (d, *J* = 7.1 Hz, 2H) ppm. ^13^C NMR (75 MHz, CD_2_Cl_2_) δ 152.3, 149.7, 149.0, 148.7, 138.6, 137.1, 134.3, 132.7, 130.6, 129.7, 129.4, 129.0, 127.4, 123.8, 121.8, 120.2, 118.3, 116.9 ppm. HRMS (ESI): *m*/*z* calcd. for C_49_H_32_IrN_6_ 897.2318; found: 906.8221 (4 + Li-H).

## 3. Results and Discussion

### 3.1. Preparation and Characterization of Complexes ***1***–***4*** and Ligands ***L1*** and ***L2***

The synthetic approach for developing bifunctional mitochondrial selective Ir(III) complexes that can act as both, PSs and cell imaging probes entails the combination of a bioactive Ir(III) yellow/green emissive fragment with a blue emissive organic chromophore (acridine or anthracene), both of them connected by 2,2′-dipyridylamine (dpa). As typically the emission of Ir(III) fragments of the type [Ir(C^N)_2_(N^N)]^+^ is highly conditioned by the nature of cyclometallated ligand, ppy and bzq will be used to fine tune the metal based optical properties.

**Scheme 1:**
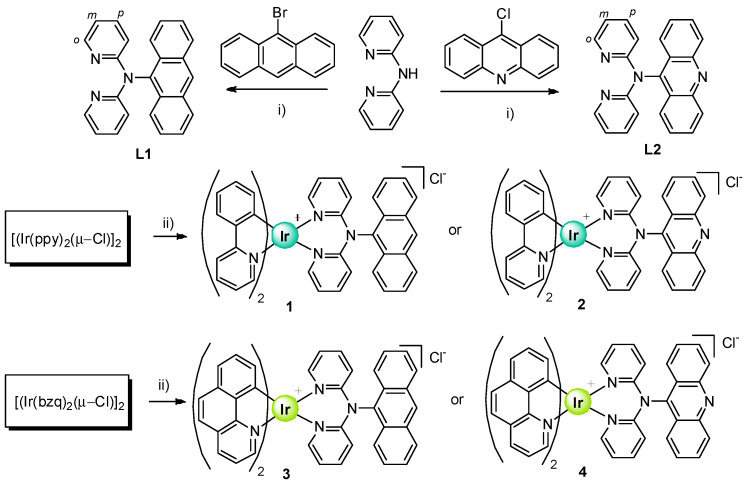
Synthetic pathway for the synthesis of **L1** and **L2** and complexes **1**–**4**. Reaction conditions as follow: (i) ^t^BuOK, toluene, reflux, 12 h; (ii) MeOH, reflux, 15 h under Ar.

Starting from the dpa and following a slightly modified procedure described by our research group [28,29], ligands **L1** and **L2** were synthesized, see Scheme 1. Specifically, by treating dpa in presence of ^t^BuOK in toluene, it was plausible to deprotonate the amine group and coordinate the correspondent chromophore, anthracene or acridine, starting from their halogen precursors. After that, the different Ir(III) complexes were obtained by refluxing together stoichiometric amounts of **L1** or **L2** and [Ir(ppy)_2_(μ-Cl)]_2_ or [Ir(bzq)_2_(μ-Cl)]_2_ in methanol. Complexes **1**–**4,** as well as ligands **L1** and **L2,** were obtained in high yield as detailed in the experimental section. Their structures were fully characterized using ^1^H and ^13^C NMR spectroscopy and mass spectrometry, corroborating the accomplishment on their synthesis (Figures S1–S12). In all cases, ^1^H NMR spectra were well defined and showed the typical patterns for the dipyridylamine derivatives coordinated to a cyclometallated Ir(III) core. Specifically, the pyridine protons of the N^N ligands shift to lower field with respect to the free ligand. Additionally, which could be appreciated in the shift of the aromatic C*H* proton in *para* position to the nitrogen of the pyridyl ring of **L2**, see Scheme 1 and Figure S3. The doublet of doublet peak at 7.65 ppm observed in the free ligand **L2**, shifted to 7.24 and 7.01 ppm in complexes **2** and **4,** respectively. Simultaneously, a set of eight resonances (each integrating as 2H) is observed for the two orthometallated ligands, due to the C_2_ symmetric nature of these complexes. Furthermore, another set of four resonances is observed for the two pyridyl rings present in the N^N ligands [30]. Similar shifts and ^1^H NMR patterns were found for complexes **1** and **3**. Additionally, the stability of the complexes was assessed by ^1^H NMR spectroscopy in DCM solution. All of them were stable for at least 48 h.

### 3.2. Optical Properties of Complexes ***1***–***4***

Photophysical properties of complexes **1**–**4** and **L1** and **L2** were measured in DMSO solution at rt. DMSO was specifically chosen for these studies to gain information of the photophysical behavior of the complexes in a polar media. Moreover, DMSO is also used to prepare the stock solutions of the complexes for the biological assays; therefore, it is a good approximation to know the photophysics of the complexes in biological media. Data collected from UV-vis absorption and emission spectroscopy are depicted in Table 1.

**Figure 3:**
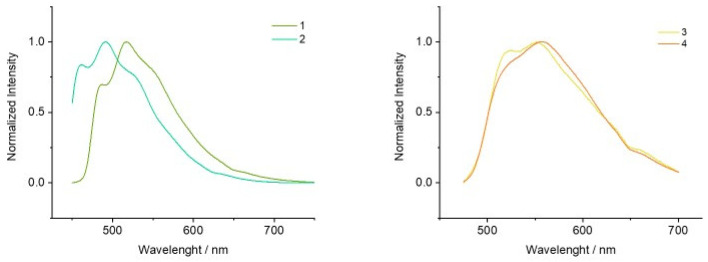
Emission spectra of complexes **1**–**4** in DMSO solution at 298 K.

Specifically, complexes **1**–**4** showed the typical absorption spectrum for cationic species of the type [Ir(C^N)_2_(N^N))]^+^, where the UV region is dominated by spin allowed ligand centered (^1^LC, π→π*) transitions and the region between 350 and 450 nm, by less intense absorption bands correspondent to ^1^MLCT and ^1^LLCT transitions, Figure S19. [31] In addition, the weak long tail observed over 450 nm is assigned to the direct excitation of spin forbidden ^3^MLCT, ^3^LLCT and ^3^LC transitions, which are taking place because of the high spin-orbit coupling of the iridium metal (Figure S19). Regarding the emissive properties, the complexes showed a strong emission band between 485 and 515 nm (**1** and **2**) and between 521 and 547 nm (**3** and **4**) (Figure 3).

**Figure 4:**
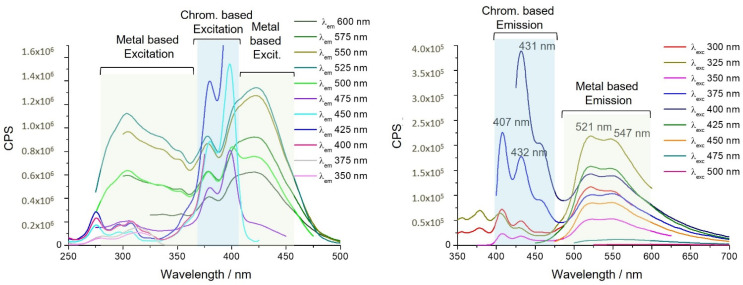
Excitation spectra (**left**) of complex **4** for the different emission profiles and the correspondent emission spectra (**right**) upon excitation at different wavelengths.

Such a small red shift observed from both families is related with the increment of the aromaticity of the cyclometallated ligand, going from a ppy in complexes **1** and **2** to a bzq in complexes **3** and **4**. Generally, the highest-occupied molecular orbital (HOMO) is and admixture of Ir dπ orbitals (t_2g_) and the π orbitals of the cyclometallated ligand, therefore higher aromaticity on the C^N ligand leads to a destabilization of the HOMO orbitals and consequently a red shift on the emission is observed [32]. The nature of the emission is associated with an admixture of ^3^MLCT and ^3^LLCT transitions for complexes **1**–**4**. Moreover, it is worth highlighting that when irradiation of complexes between 375 and 400 nm an additional high energetic emissive band is observed; see Figure 4 and Figures S15–S18. The new band has a structured pattern that resembles that of **L1** and **L2** and, specifically, that of the anthracene and acridine chromophores (Figures S13 and S14). Such emission band has its maximum intensity when the excitation is taking place around 400 nm, and it can be attributed to a ^1^LC transition within the chromophore. This secondary emission has a higher contribution in the case of complexes **3** and **4**, possibly due to the fact that the emission derived from the triplet state is red shifted (c.a. 521 nm) in comparison with that of complexes **1** and **2** (c.a. 486 nm) and therefore, facilitating its detection. Figure 4 shows the emissive and excitation profile of complex **4** when irradiated at different excitation wavelengths, demonstrating that upon selection of the excitation wavelength, the main emission displayed by the species can be shifted, from a chromophore based to an Ir-based emission. Similar analysis is depicted in the supporting information in Figures S15–S17 for complexes **1**–**3**. Regarding the lifetimes of the excited state, all complexes presented a lifetime value between 186–456 ns for the iridium-based emission, similar to that of [Ir(ppy)_2_(bpy)]PF_6_ [33] and indicating the phosphorescence nature of the process. Additionally, quantum yields were registered in aerated DMSO, revealing values between 6.5 and 10%.

### 3.3. Antiproliferative Activity of Complexes ***1***–***4***

The sensitivity of A549 lung carcinoma cells to **1**–**4** and **L1** and **L2** was examined by MTT-reduction assays after 24 h of treatment. Data gathered in Table 2 reveals that complexes **1**–**3** displayed a moderate antiproliferative activity, with IC_50_ values between 13.6 µM and 19.6 µM followed from afar by compound **4**, which yielded a IC_50_ value of 43.38 µM. Conversely, high IC_50_ values obtained for both ligands involves that they did not perturbed cell viability at the studied concentration range. These results therefore suggest that the bioactivity falls to the metallic fragment. Alternatively, additional photosensitivity studies were performed using an irradiation wavelength of 405 and 470 nm to elucidate whether these complexes could be used as PSs as well as the optimum excitation wavelength. Thus, A549 cells were incubated with complexes for 5 h, for 10 min at either 405 nm or 470 nm and kept in culture for a further 19 h. Despite reducing the IC_50_ values from 7 to 14 times when irradiation at 405 nm was performed, it is actually the irradiation at 470 nm when the greatest antiproliferative effect is detected for all complexes. The IC_50_ values for all the complexes dropped to the nanomolar range. Among all them it is striking the behavior of complex **4** as cells showed little sensitivity to this complex under no irradiation conditions (43.38 ± 0.14 μM) but they were extremely sensitive when irradiated at 470 nm, reducing its IC_50_ value more than 110 times and matching its antiproliferative capacity to compounds **2** and **3**. Keeping in mind that good PSs should only reveal anticancer properties under irradiation conditions, compound **4** is proposed to be the best PS agent from the series complexes **1**–**4**. On the other hand, the marked difference in bioactivity offered between the two irradiation wavelengths is probably due to the diverse nature of the excited state when irradiating at 405 or at 470 nm. Irradiation of the complexes at 405 nm is mainly affecting the organic chromophore. On the contrary, irradiation at 470 nm excites exclusively the iridium fragment, which by means of the heavy atom effect evolves effectively to a triplet excited state, with longer excited state lifetimes and affording better PS source for photodynamic therapy.

### 3.4. Cell Morphology

**Figure 5:**
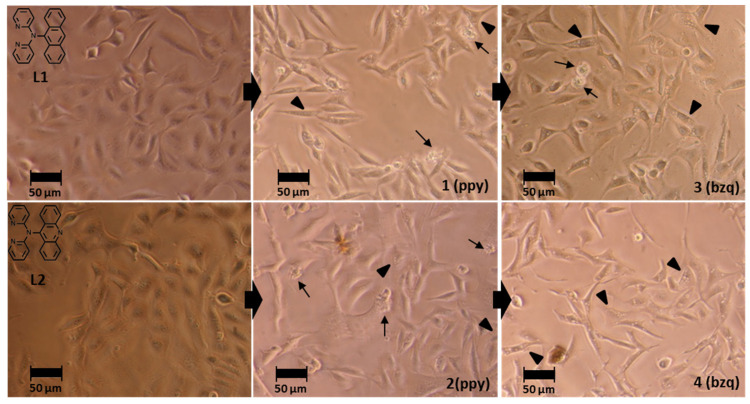
Phase contrast microscopy images of A549 cells treated with **L1**, **L2** and **1**–**4** at concentrations between 10 and 20 µM for 20 h. Black arrows point to apoptotic cells. Little black triangles show examples of cells containing cytoplasmic vesicles.

Alterations in cell morphology and behavior as a consequence of the exposition to ligands and complexes were analyzed using an inverted microscope (Figure 5). As expected, healthy cells displayed an epithelial-like morphology, and they grew exponentially (data not shown). A similar appearance was observed when treating cells with **L1** and **L2** as several dividing cells were discerned and apoptotic or necrotic cells were not detected. Oppositely, complexes **1**–**4** were able to modify cell aspect. All of them triggered cytoplasmic vesicle formation, which was more remarkable for compounds **3** and **4**, containing the bzq orthometallated ligand. Moreover, even though cell division still took place in the presence of all of them, it became patently clear that they have cytotoxic properties, being more evident for complexes **1** and **2**.

### 3.5. Cytotoxic Activity, Cell Cycle Arrest of Complex 4 and Partition Coeficient Octanol Water for ***1***–***4*** and ***L1***–***L2***

Previously to cytotoxicity assays, cells were visualized under an inverted microscope in order to observe morphological changes. As expected, control cells were growing exponentially but treatments provoked large cytoplasmic vacuolization and apoptotic and necrotic morphologies (data not shown).

**Figure 6:**
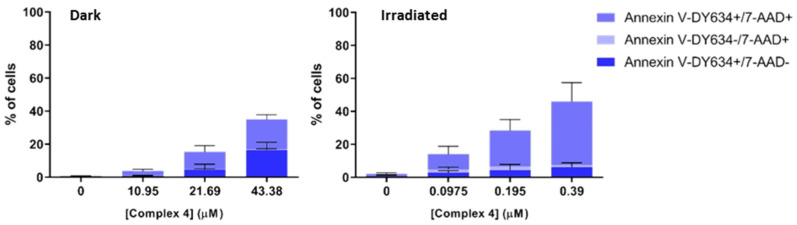
Cytotoxicity assays of complex **4**, when it was incubated in A549 cell line for 24 h, in a range of concentration between its IC_50_ and ¼ IC_50_ values. In dark (**left**); Under irradiation at 470 nm (**right**).

Cytotoxic potential of complex **4** was analyzed by flow cytometry using the simultaneous double staining of Annexin V-DY634 and 7-AAD in both, dark and under irradiation conditions (λ_exc_: 470 nm). Dead cells that preserve cell membrane integrity exhibit phosphatidylserine on cell surface. This exposition is a typical feature of ‘early apoptosis’ and it can be detected by flow cytometry thanks to protein Annexin V conjugated with different fluorophores, which selectively binds to this phospholipid in the presence of calcium [34]. However, cells with compromised cell membrane (necrotic cells) allow DNA labeling with fluorescent intercalating agents such as 7-AAD. Furthermore, cells can be double stained by undergoing “late apoptosis” as they initiate the apoptotic process and end up suffering secondary necrosis upon loss of membrane integrity. As shown in Figure 6, complex **4** displayed a concentration-dependent cytotoxic activity in both conditions. Although in both cases the death of the cells was induced, the way in which they died differs. When cells were not irradiated, they underwent early and late apoptosis, with the late state predominating at low dose and reaching a similar distribution between both states at high concentrations. However, irradiated cells mostly reached the late apoptotic state although apoptotic cells at the early state were also detected. Therefore, these results suggest that complex **4** triggers the apoptotic pathway in A549 cells under dark and irradiation conditions. In addition, since massive cytoplasmic vacuolization is a hallmark of paraptosis [35], we assume that this type of programmed cell death coexists with apoptosis though more exhaustive studies are necessary to confirm this hypothesis.

**Figure 7:**
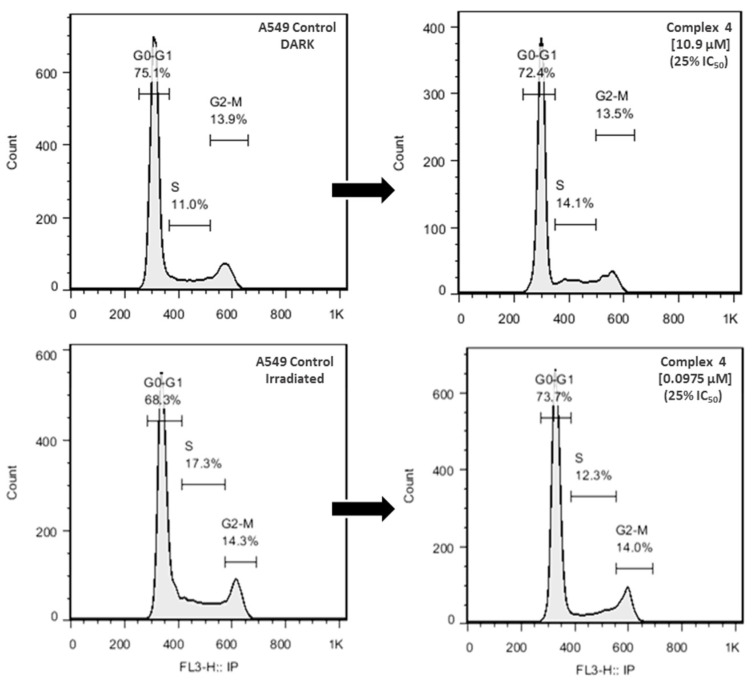
Cell cycle analysis after treatment with complex **4** in dark (**top graphs**) and under irradiation at 470 nm (**bottom graphs**). The cell distribution at each phase is indicated for each graphic.

To delve into the mechanism of action of the complex, we decided to evaluate its ability to modify cell cycle. The cell cycle is a vital process for cells as it implies their replication and thus, the survival of the cells. Biologically wise is an ordered series of events involving cell growth and cell division to eventually produce two identical cells [36]. Specifically, four different phases define the cell cycle: cells grow and are metabolically active throughout G1 phase, they duplicate their DNA content during S phase, prepare for cell division in the course of G2 phase and finally they divide resulting in two daughter cells during M phase, each of which will carry out its own cell cycle. G1, S and G2 phases in turn constitute the interphase, the stage of the cell cycle where cells spend most of the time. Cell cycle was analyzed in presence of complex **4** in dark and upon irradiation conditions. The objective of this experiment is to elucidate if the increment of antiproliferative potential upon irradiation of complex **4** at low concentrations could be related with its ability of inducing cell cycle arrest at any of its phases, which might be different from its behavior in dark conditions. Figure 7 indicates that untreated cells in dark conditions and upon irradiation at 470 nm for 10 min behave similarly. In both cases they present the typical cell cycle pattern for healthy cells, where most of them are growing exponentially at the G0/G1 phase. In addition, the cell distribution at the different phases is hardly disturbed with respect to their respective controls upon the different treatments. Consequently, the results obtained seem to indicate that complex **4** is not capable of stopping the cell cycle at any of its phases in both under dark and irradiation conditions.

Additionally, the ratio lipophilicity: hydrophilicity can be measured by the partition coefficient water/n-octanol, logP, by experimental procedures such as the shake flask method among others [37]. The value of logP indicates the distribution of the drug between an equal amount of n-octanol and buffered aqueous solution. In early drug discovery, assessing the lipophilicity of a potential drug is essential to predict to it physicochemical behavior in vivo, and it is one of the parameters use in Lipinky rule for assessing the druglikeness of a compound [38]. Table 3 displays logP for all the complexes as well as **L1** and **L2**. Thus, logP values ranges from 0.28 in the case of complex **2**, being the less lipophilic, to 1.73 for complex **3** at the other far end. These results are in concordance with intrinsic higher lipophilicity of **L1** vs. **L2** added to the more lipophilic nature of the orthometalated bzq in complexes **3** and **4** vs. ppy in **1** and **2**.

### 3.6. Subcellular Localization of Complexes ***1***–***4***

**Figure 8:**
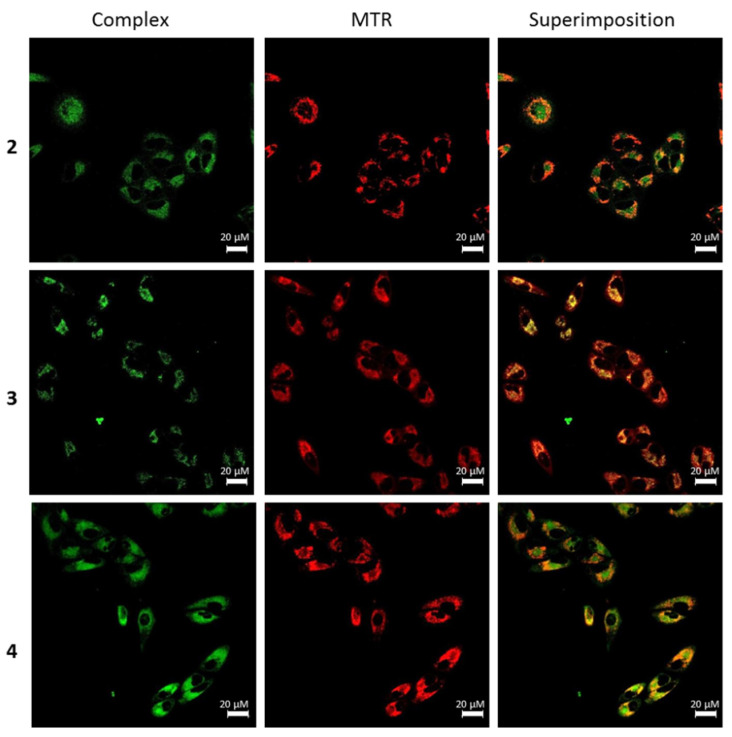
Fluorescence confocal microscopy images in A549 cells incubated with complexes **2**, **3**, **4** (green, irradiated at 458 nm) and stained with MTR (red, irradiation at 598 nm).

Confocal microscopy assays were carried out to elucidate the biodistribution of complexes **2**–**4** in A549 cells, and to further assess the possibility of targeting mitochondria due to their great importance as biological targets in PDT [15,17]. Therefore, a colocalization assay was performed where complexes **2**–**4** were incubated with A549 cells together with a commercially available mitochondrial selective dye (MitoTracker-red, MTR). Figure 8 shows slightly different emission patterns for each complex. While complex **2** and especially **3** display a punctate emission pattern, that emitted by complex **4** is more diffuse. In any case, complex **3** fully colocalizes with the signal emitted by MTR indicating its mitochondrial localization, whereas complexes **2** and **4** do so partially.

**Figure 9:**
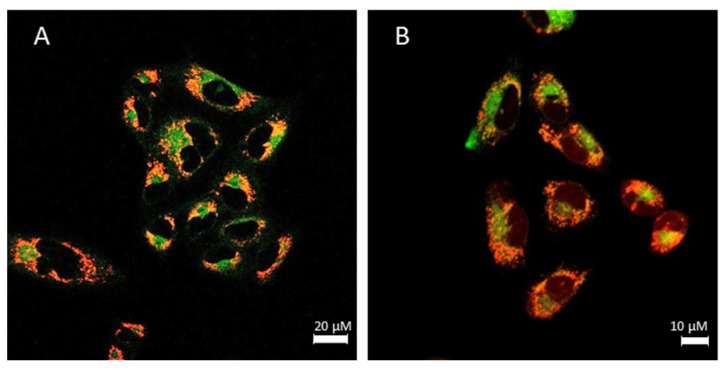
Superimposition images in A549 cells of complexes **2** (**A**) and **4** (**B**) incubated 2 h and stained with MitoTracker Red (MTR).

A closer look to Figure 9 also suggests that, apart from a partial mitochondrial localization, complexes **2** and **4** have an additional biodistribution close to the nuclear region. This distinct pattern was not seen for complex **3** and it might be related to the different coupled chromophore, i.e., acridine for **2** and **4** and anthracene in the case of complex **3.** The nitrogen present in the acridine is in fact, the only structural difference between the complexes. Recently Zhao, Zhang and coworkers also described a mitochondrial selective Ir(III) complex containing an anthracene moiety [39] and, once again, suggesting the role that the chromophore might be exerting on the biodistribution behavior. On the contrary, the distribution of the complexes in this case might point to a localization in the Golgi apparatus, since the cisternae of the complex are usually found together and near the nuclear region [40]. In fact, some Ir(III) compounds have been described that accumulate in the Golgi apparatus [41,42]. Notwithstanding, further studies are required to fully clarify the distribution of complexes **2** and **4** within cells.

### 3.7. ROS Generation Potential

**Figure 10:**
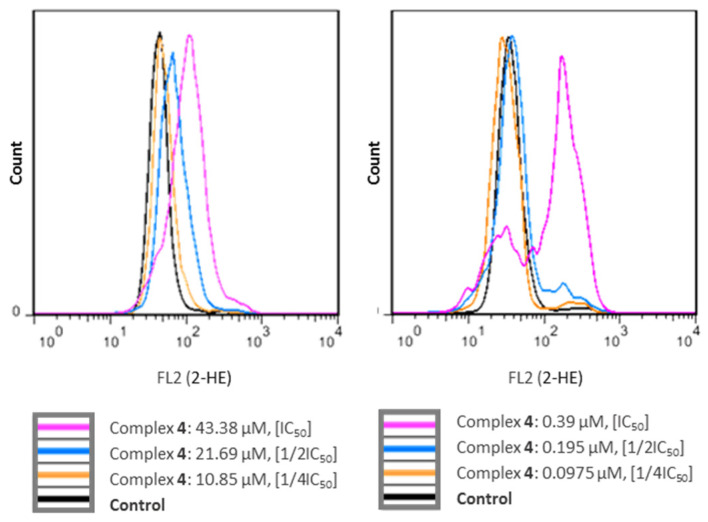
Generation of ROS induced by complex **4** at increasing concentrations in dark (**left graph**) and upon irradiation at 470 nm (**right graph**).

After seeing that the PI of **4** is 111.4 times and knowing that such increment could be driven by the generation of ROS species as a consequence of phototherapeutic effect that the probe might exert upon irradiation, an assessment on the generation of ROS was undertaken by flow cytometry using dihydroethidium (DHE) as a fluorescent probe. The DHE probe is oxidized in presence of the superoxide ion to 2-HE (2-hydroxyethidium). The assay was performed once again in dark and upon irradiation conditions and the result is depicted in Figure 10. Thus, complex **4** showed a concentration-dependent ROS generation in both assay conditions and ROS production was greater when the complex at high doses was irradiated at 470 nm for 10 min. Interestingly, each condition yielded different ROS production profiles. When the compound was not irradiated, a single peak shifted to the right is observed, as a sign of an increase in ROS generation at a general level; in contrast, when irradiating at high doses, two peaks are observed, one shifted to the right and the other remaining at the control level. This implies that a fraction of the cells has overactivated their ROS production mechanism whereas the rest maintain production levels similar to those of untreated cells. These high levels of ROS might be a consequence of the activation of the iridium complex that enables to reach the iridium complexes a triplet excited state. At this point an electron transfer process (PDT type I) and/or energy transfer process (PDT type II) takes places generating the highly cytotoxic ROS [43]. In conclusion, A549 cells enhanced ROS production after exposition to non-irradiated or especially to irradiated complex **4**.

## 4. Conclusions

Four new luminescent and photosensitive iridium(III) complexes of the type [Ir(C^N)_2_(N^N-R)]^+^, where N^C is either phenylpyridine (ppy) or benzoquinoline (bzq), N^N is 2,2’-dipyridylamine (dpa) and R either acridine or anthracene, have been synthesized and their emissive and bioactivity properties against A549 cell line have been studied. The optical analysis showed an excitation dependent emissive pattern for all the complexes which was more evident in the case of complex **4**. Specifically, complex **4** clearly showed that the main emissive band was due to the organic chromophore (LC transition) when the irradiation was performed between 374 and 400 nm with a structured band between 407 and 450 nm, whereas irradiating at different wavelength ranges, the main emission was centered c.a. 525 nm, and it was due to the iridium fragment (^3^MLCT and ^3^LLCT transition). The difference on the emissive origin (acridine vs. Ir(III) fragment) was reflected in the photocytotoxic properties observed upon incubation with A549 cells All the complexes displayed moderate antiproliferative activity, being complex **4** the least potent. However, under irradiation conditions their bioactivity increases especially when the irradiation affects the Ir(III) core. Thus, complex **4** has an IC_50_ value of 43.38 ± 0.14 μM in dark, IC_50_ > 5 μM upon irradiation for 10 min at 405 nm and IC_50_ of 0.39 ± 0.09 μM upon irradiation for 10 min at 470 nm. These results emphasize the importance of the intrinsic photophysical properties of Ir(III) core over that of organic chromophores for delivering upgraded PSs. Microscopy analysis revealed cytoplasmic vacuolization and typical apoptotic and necrotic features which were confirmed by cytotoxicity assays, suggesting that complex **4** prompts paraptotic and apoptotic cell death. In fact, we have previously described several heterometallic Ir(III)-Au(I) compounds that appear to activate the paraptotic pathway first and the apoptotic pathway ultimately [14]. Confocal fluorescence microscopy studies propose that complex **3** entirely localized in mitochondria, and compounds **2** and **4**, although also partly localized in these organelles, present other subcellular distribution. As both complexes (**2** and **4**) contain the same chromophore (acridine) it can be suggested that the chromophore’s role within subcellular localization is not innocent. However, further biodistribution assays need to be performed prior to stablish any structure/localization relationship. Moreover, no cell cycle alterations were detected but a greater ROS generation was observed under dark and irradiation conditions. These complexes are the proof of concept that it is possible to deliver dual emissive Ir(III) probes as effective PSs for PDT and cell imaging agents. Despite the promising results, further structural modifications are needed to obtain future agents that absorb in the red or near infrared range, the phototherapeutic window.

## Figures and Tables

**Table 1:** Photophysical data of **L1**, **L2** and **1**–**4** measured in aerated DMSO solution.

	UV-vis ^a^ (×10^3^ ε/dm^3^ mol^−1^ cm^−1^)/nm	λ_em_ [λ_exc_] (nm) ^b^	τ (ns) ^b^	Φ (%) ^c^
**L1**	260 (47.5), 318 (8.7), 355 (3.4), 373 (5.2), 394 (4.5)	405, 422, 448 [375]	-	-
**L2**	260 (47.5), 270 (30.7), 317 (16.2), 350 (7.5), 362 (9.8), 388 (5.4)	409, 431, 557 [400]	-	-
**1**	260 (94.4), 320 (14.5), 354 (10.0), 375 (10.7), 395 (8.9), 424 (3.9)	486, 515s, 551 [437]	186	8.3
**2**	260 (87.5), 273 (68.1), 310 (25.4), 380 (20.2), 400 (22.1), 427 (6.5)	485, 514 [433]	210	8.4
**3**	260 (43.4), 324 (9.4), 353 (6.7), 374 (5.5), 394 (3.9), 423 (1.8)	521s, 551s [432]	242	6.4
**4**	260 (39.1), 271 (35.1), 308 (12.9), 380 (10.1), 398 (11.5), 425 (3.2)	521s, 547s 407s, 432, 458 [450]	451	10

^a^ in DMSO solution: **L1** and **L2** at [2·10^−5^ M] and complexes **1**–**4** at [10^−5^ M]. ^b^ In aerated DMSO solution. ^c^ In aerated DMSO at [2.5·10^−5^ M].

**Table 2:** IC_50_ values of complexes **1**–**4** and **L1**–**L2** incubated for 24 h in A549 cells.

Complexes	IC_50 (DARK)_	IC_50(λexc 405 nm)_^1^	PI	IC_50(λexc 470 nm)_^1^	PI
**L1**	>50	-		-	
**L2**	>50	-		-	
**1**	13.6 ± 0.08	1.16 ± 0.06	11.7	0.18 ± 0.05	75.5
**2**	19.6 ± 0.68	2.54 ± 0.08	7.7	0.34 ± 0.06	57.6
**3**	18.9 ± 0.12	1.35 ± 0.08	14.0	0.40 ± 0.08	47.2
**4**	43.38 ± 0.14	>5		0.39 ± 0.09	111.2

^1^ IC_50_ values in µM, irradiation time: 10 min at 405 or 470 nm after 5 h incubation. (PI: Phototoxic index).

**Table 3:** logP values of complexes **1**–**4** at pH = 7.4.

Ligands	logP(Oct/W)	Complexes	logP(Oct/W)	Complexes	logP(Oct/W)
**L1**	1.20	**1**	0.75	**3**	1.73
**L2**	0.59	**2**	0.28	**4**	1.24

## Data Availability

Data is contained within the article or supplementary material.

## Supplementary Materials

The following are available online at https://www.mdpi.com/article/10.3390/pharmaceutics13091382/s1, Figure S1: ^1^H-NMR spectrum of compound **L1**, Figure S2: APT-NMR spectrum of compound **L1**, Figure S3: ^1^H-NMR spectrum of compound **L2**, Figure S4: APT-NMR spectrum of compound **L2**; Figure S5: ^1^H-NMR spectrum of complex **1**, Figure S6: APT-NMR spectrum of complex **1**, Figure S7: ^1^H-NMR spectrum of complex **2**, Figure S8: APT-NMR spectrum of complex **2**, Figure S9: ^1^H-NMR spectrum of complex **3**, Figure S10: APT-NMR spectrum of complex **3**, Figure S11: ^1^H-NMR spectrum of complex 4, Figure S12: APT-NMR spectrum of complex **4**, Figure S13: Emission-spectra of **L1** measured in DMSO solution, Figure S14: Emission-spectra of **L2** measured in DMSO solution, Figure S15: Emission-spectra of **1** measured in DMSO solution, Figure S16: Emission-spectra of **2** measured in DMSO solution, Figure S17: Emission-spectra of **3** measured in DMSO solution, Figure S18: Emission-spectra of **4** measured in DMSO solution, Figure S19: Absorption spectra in DMSO solution, Figure S20: Fluorescence confocal microscopy images of A549 cells incubated with **2** (2 h) and stained with MTR, Figure S21: Fluorescence confocal microscopy images of A549 cells incubated with 3 (2 h) and stained with MTR, Figure S22: Fluorescence confocal microscopy images of A549 cells incubated with **4** (2 h) and stained with MTR.
