# Supplementary material for: Dual Emissive Ir(III) Complexes for Photodynamic Therapy and Bioimaging
Source: Pharmaceutics. 2021 Sep 1;13(9):1382. doi: 10.3390/pharmaceutics13091382 (PMC8472790; doi:10.3390/pharmaceutics13091382)
Supplement: Supplementary file 1 [file pharmaceutics-13-01382-s001.zip › pharmaceutics-1338408-supplementary.pdf]

pharmaceutics

MDPI

Suplementary Materials: Dual Emissive Ir(III) Complexes for Photodynamic Therapy and Bioimaging

Marta Redrado, Andrea Benedi, Isabel Marzo, M. Concepción Gimeno and Vanesa Fernández-Moreira

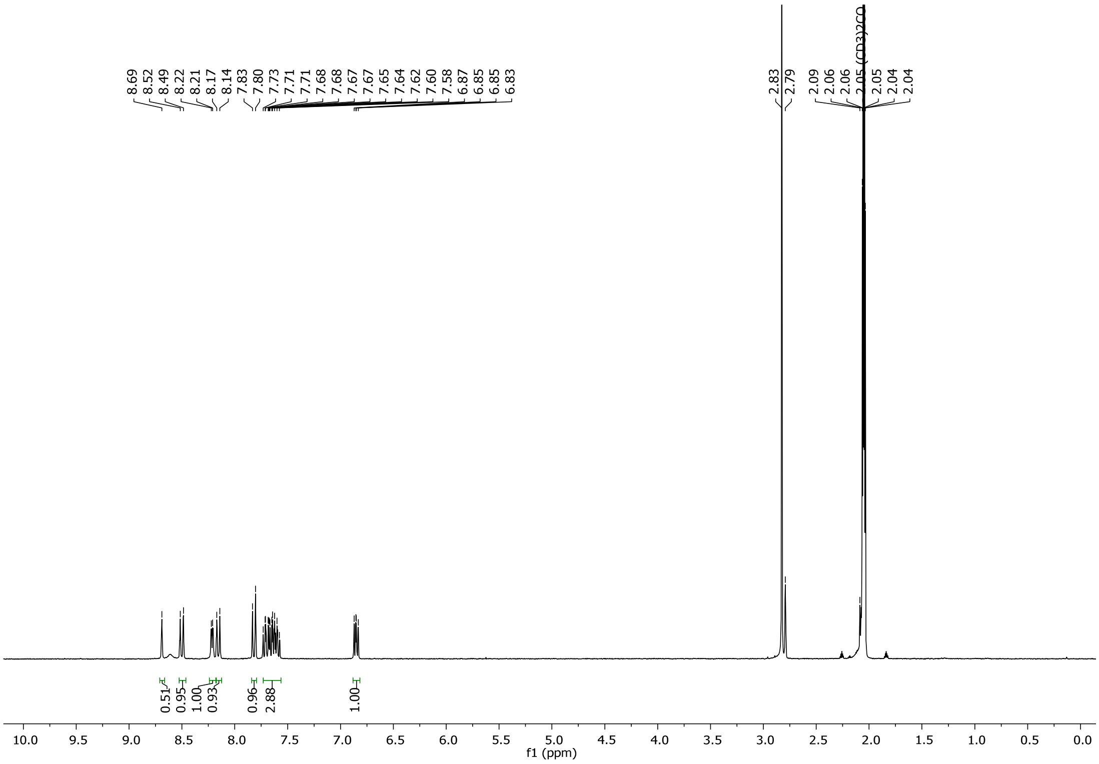

Figure S1. ^{1}H-NMR spectrum of compound **L1**.

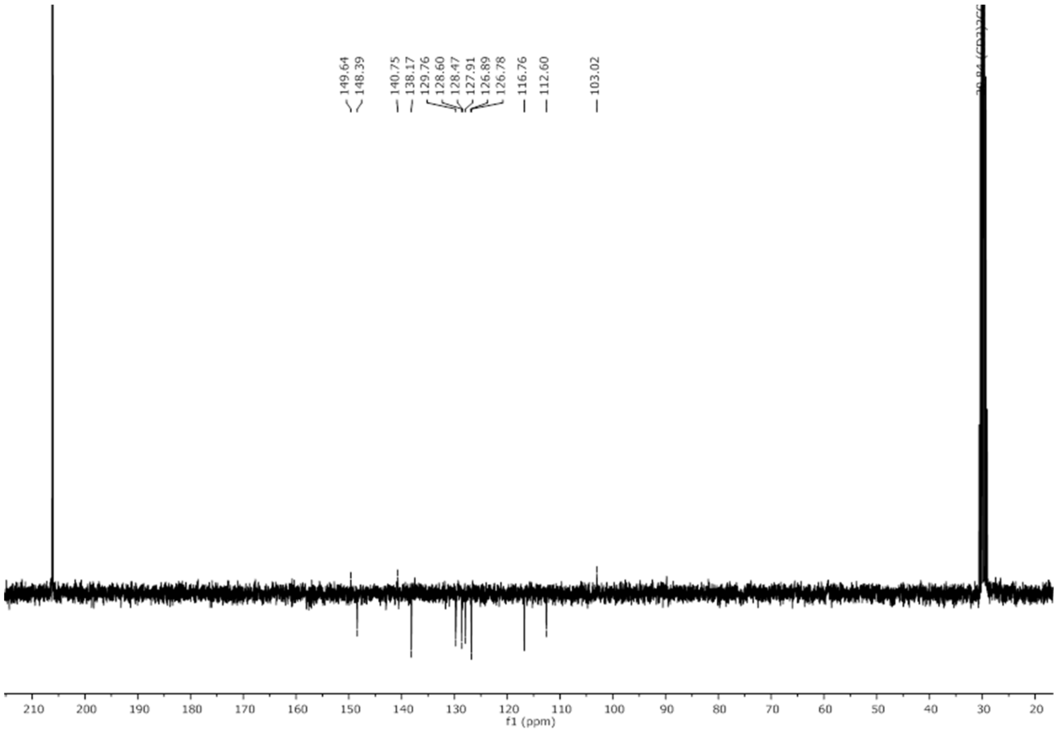

Figure S2. APT-NMR spectrum of compound **L1**.

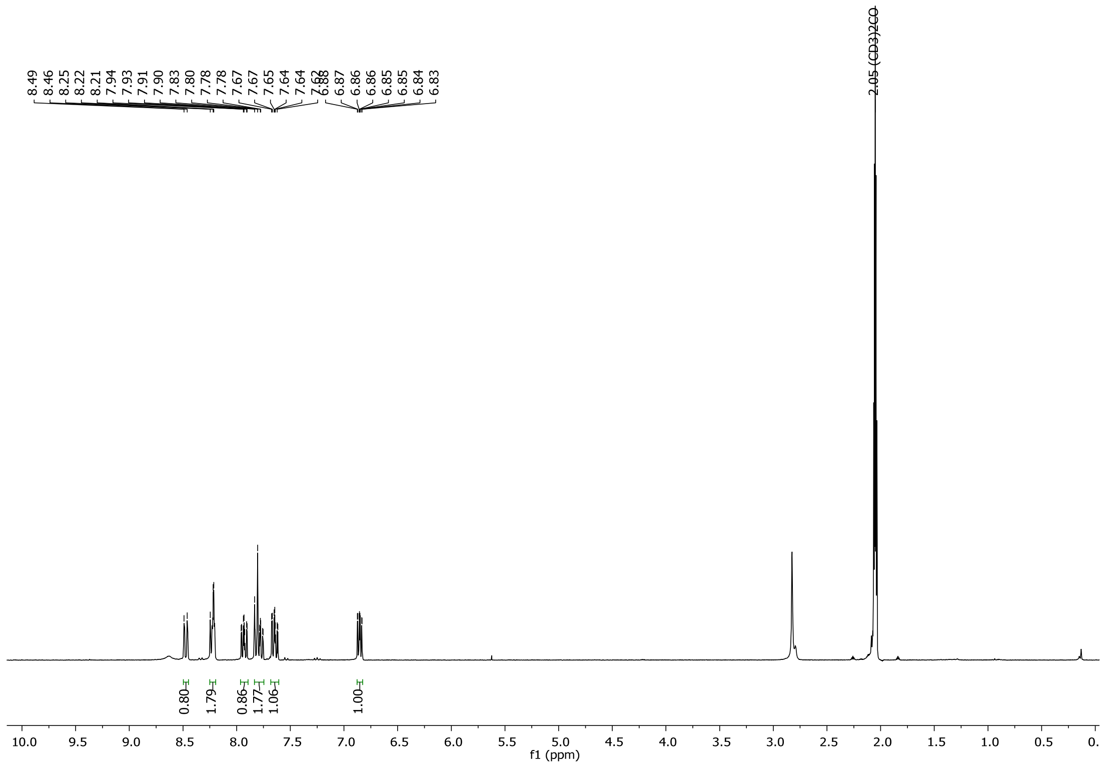

Figure S3. ^{1}H-NMR spectrum of compound **L2**.

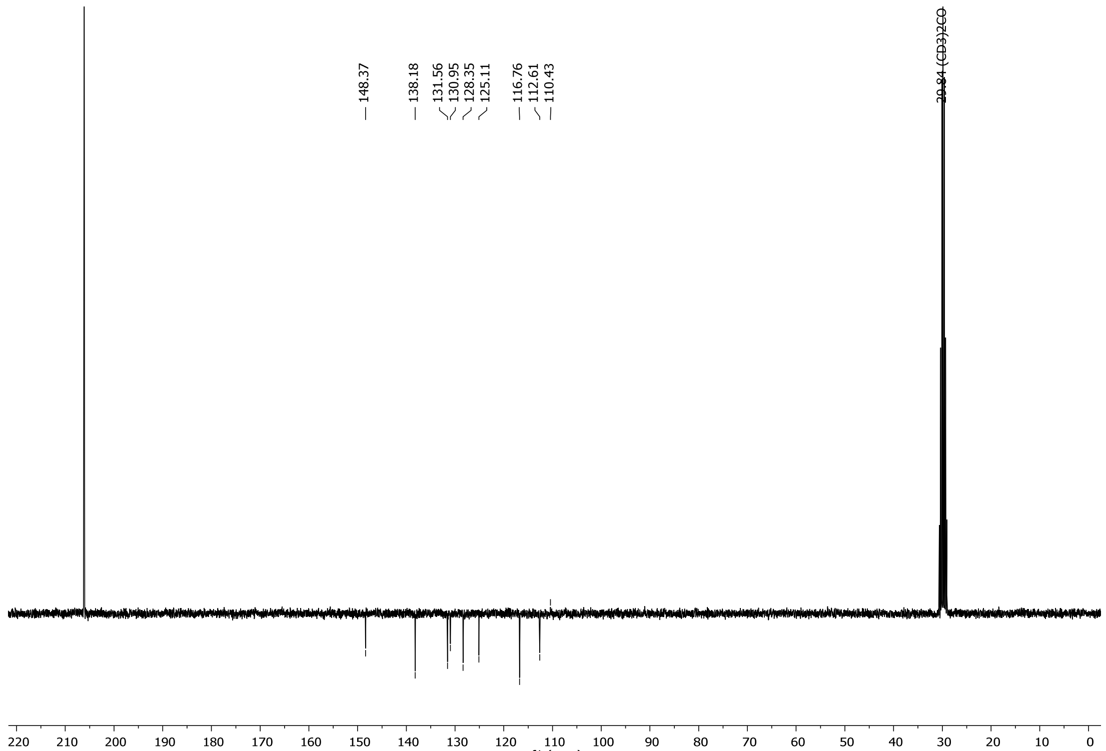

Figure S4. APT-NMR spectrum of compound **L2.**

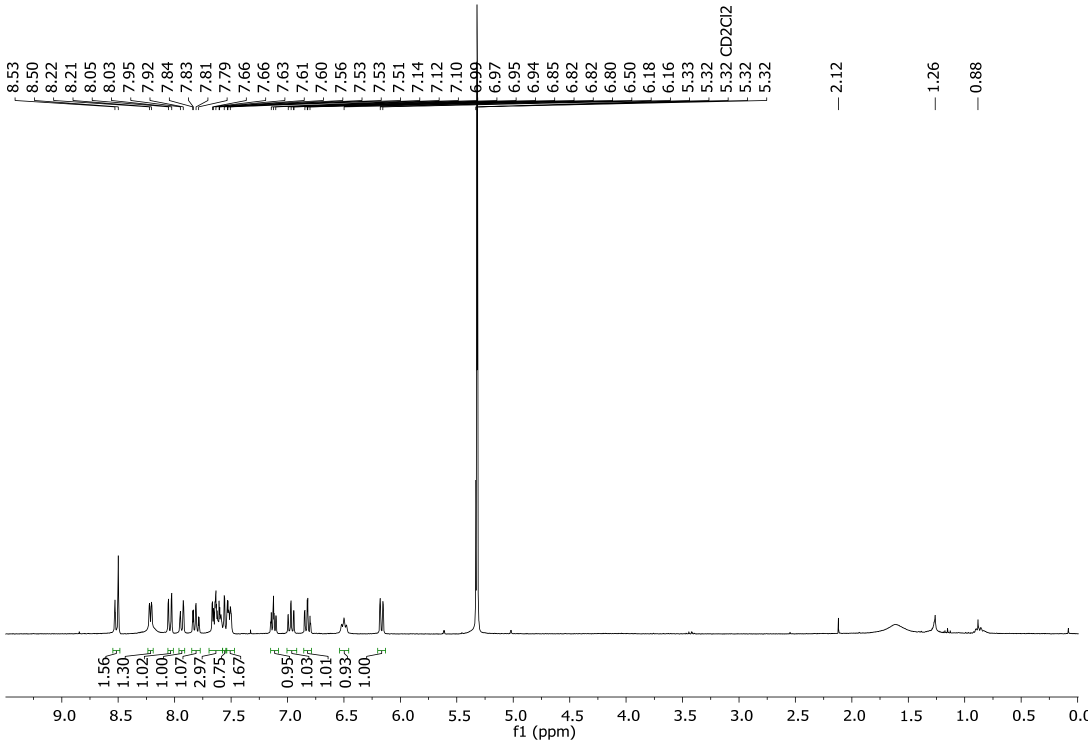

Figure S5. ^{1}H-NMR spectrum of complex **1**.

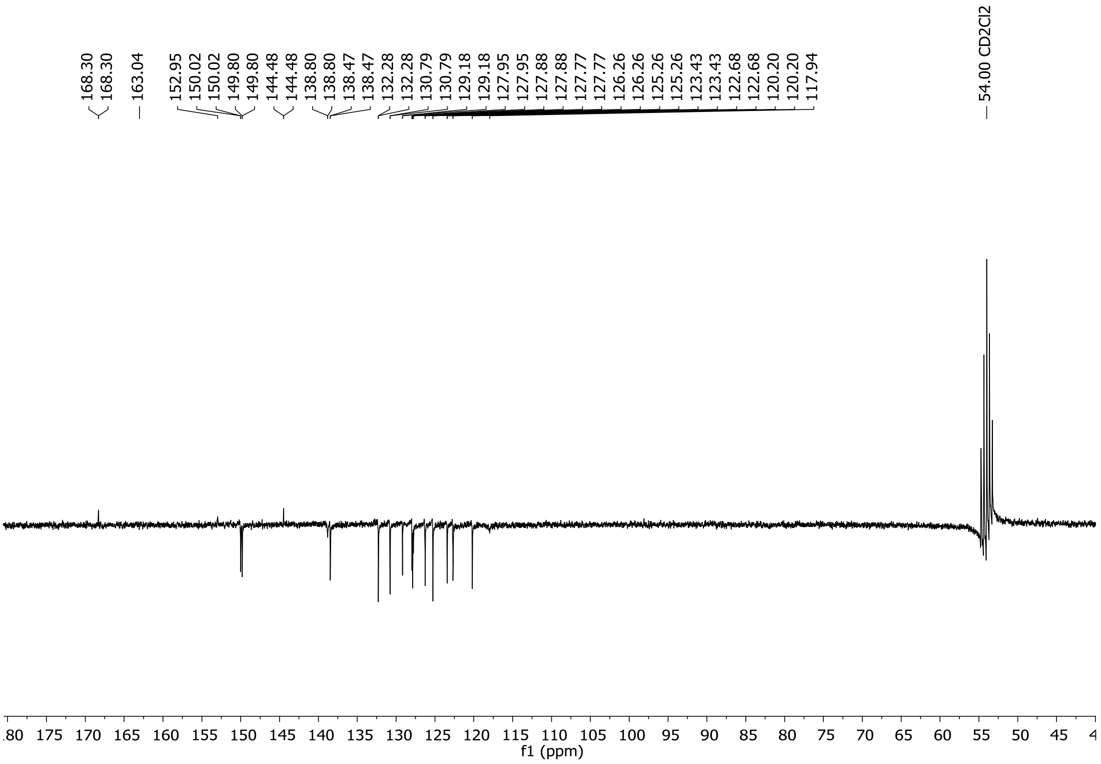

Figure S6. APT-NMR spectrum of complex **1.**

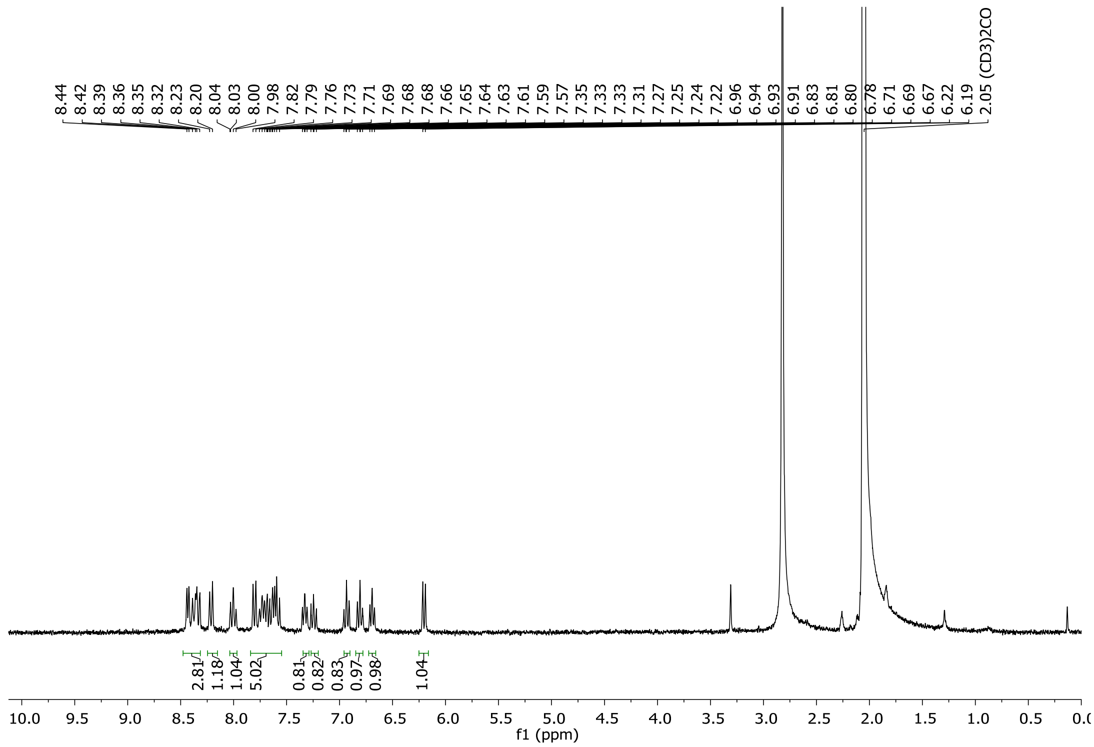

Figure S7. ^{1}H-NMR spectrum of complex **2**.

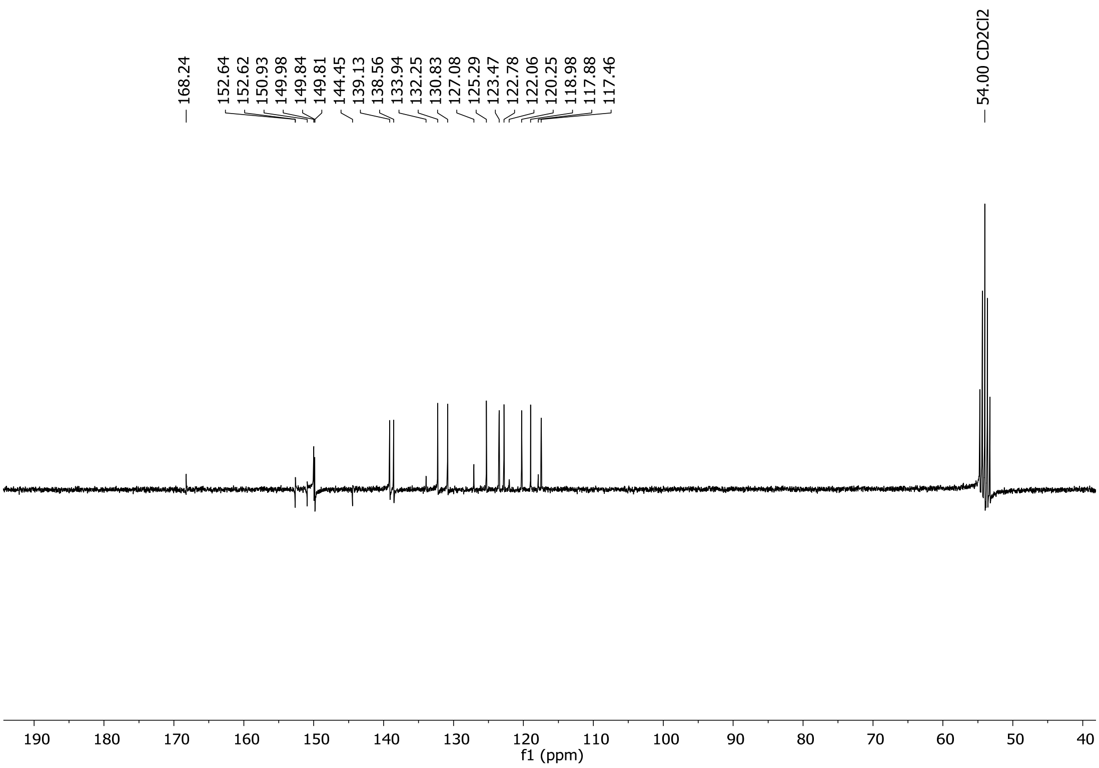

Figure S8. APT-NMR spectrum of complex **2**.

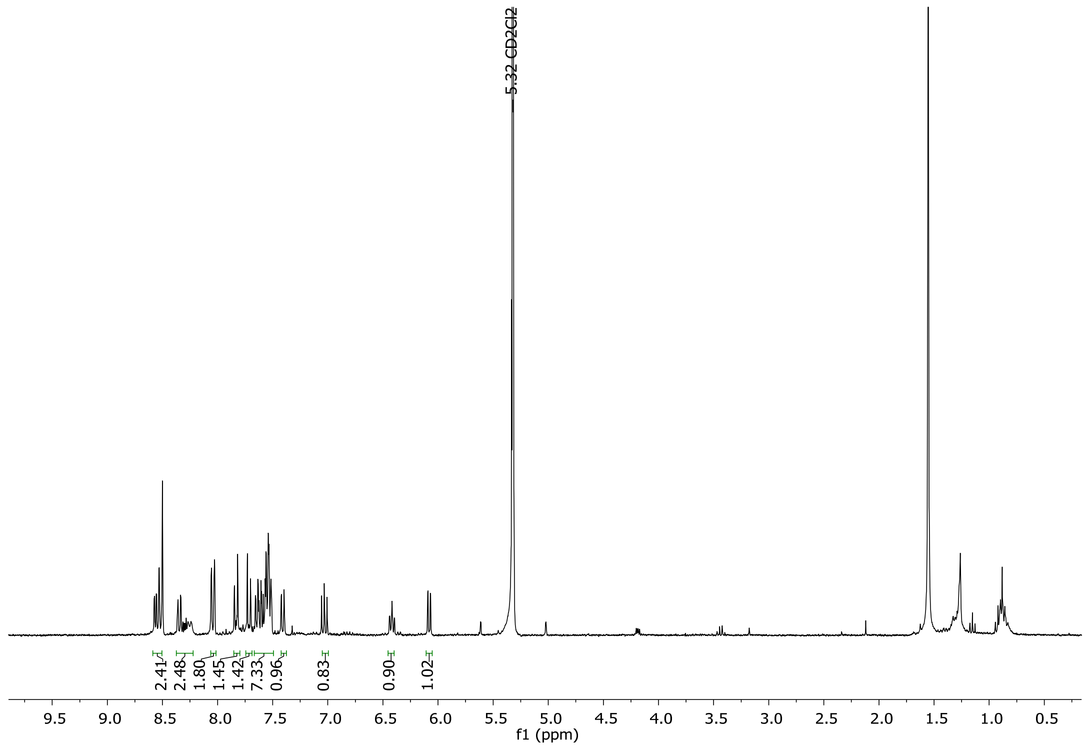

Figure S9. ^{1}H-NMR spectrum of complex **3**.

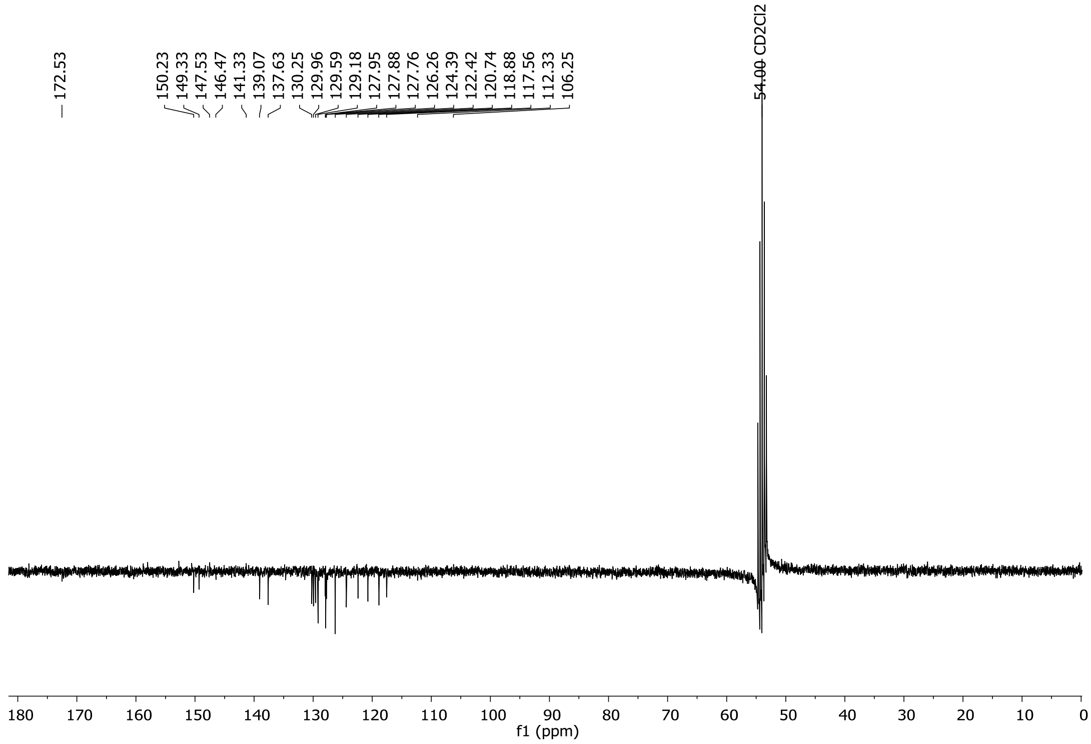

Figure S10. APT-NMR spectrum of complex **3**.

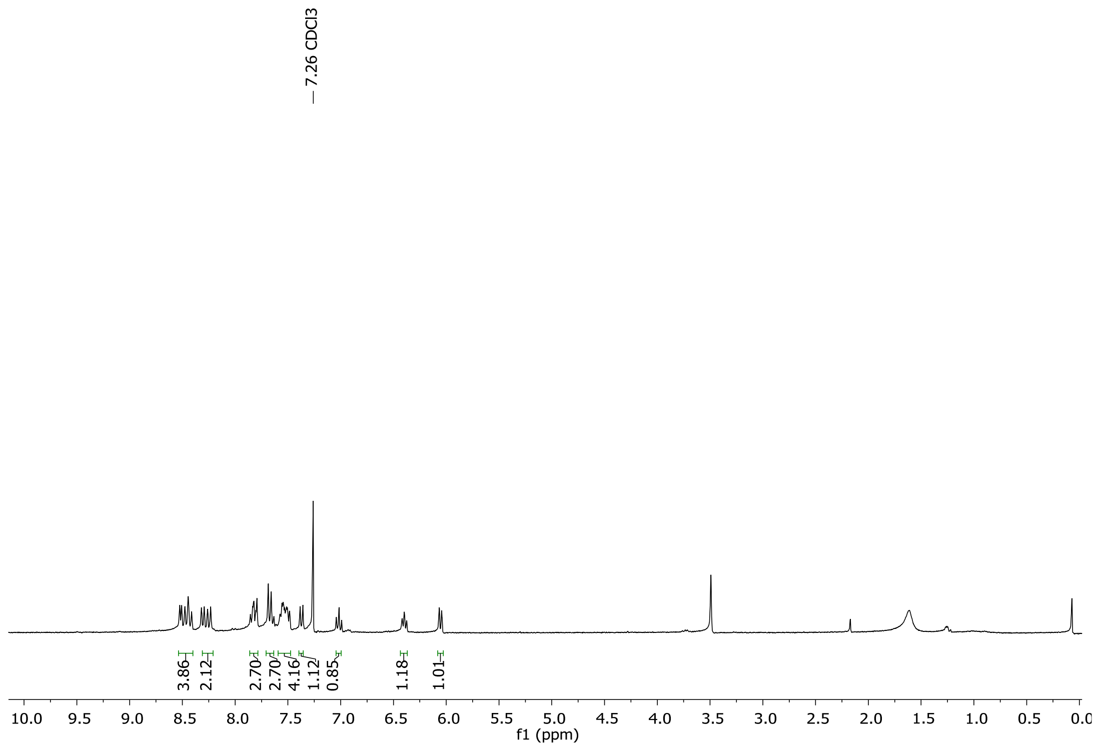

Figure S11. ^{1}H-NMR spectrum of complex **4**.

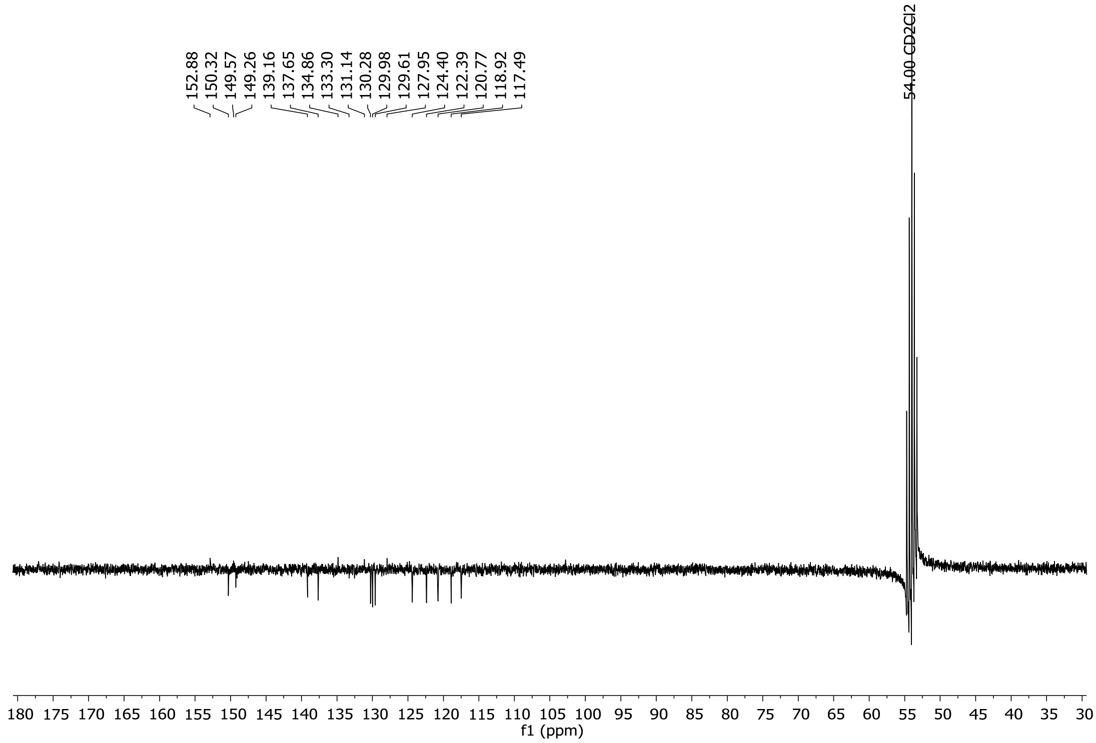

Figure S12. APT-NMR spectrum of complex **4**.

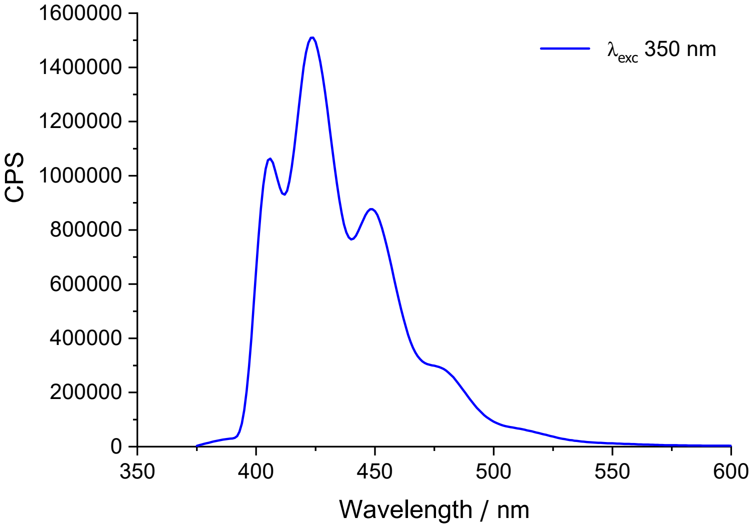

Figure S13. Emission-spectra of **L1** measured in DMSO solution.

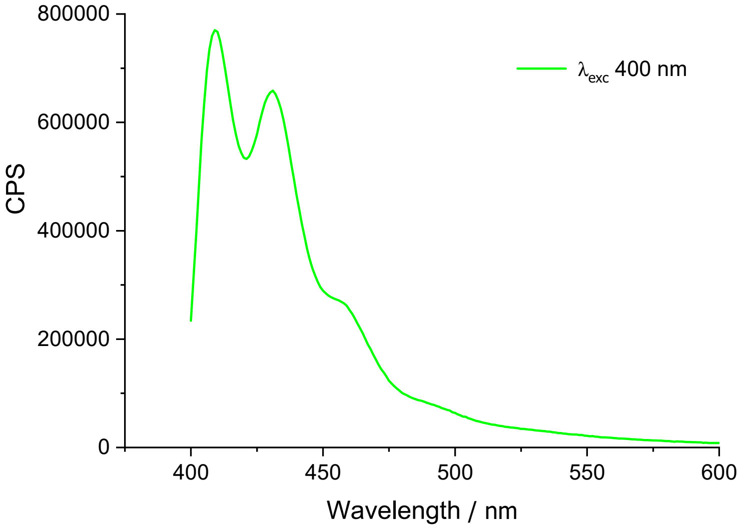

Figure S14. Emission-spectra of **L2** measured in DMSO solution.

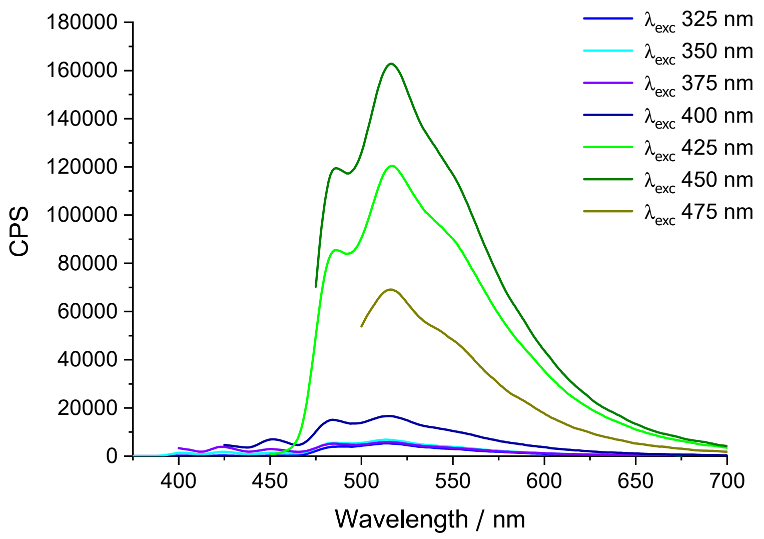

Figure S15. Emission-spectra of **1** measured in DMSO solution.

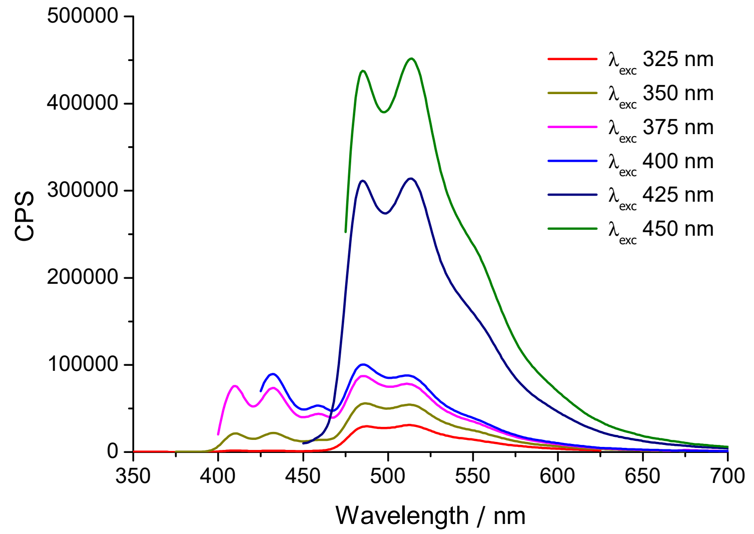

Figure S16. Emission-spectra of **2** measured in DMSO solution.

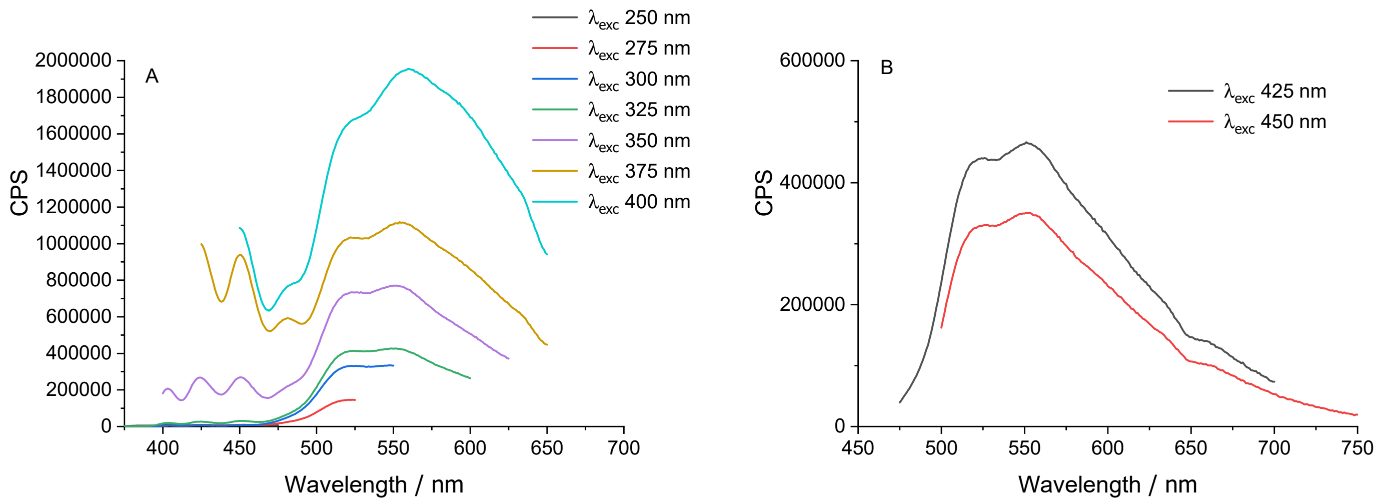

Figure S17. Emission-spectra of **3** measured in DMSO solution. A) Slit: 5nm, B) Slit: 3nm.

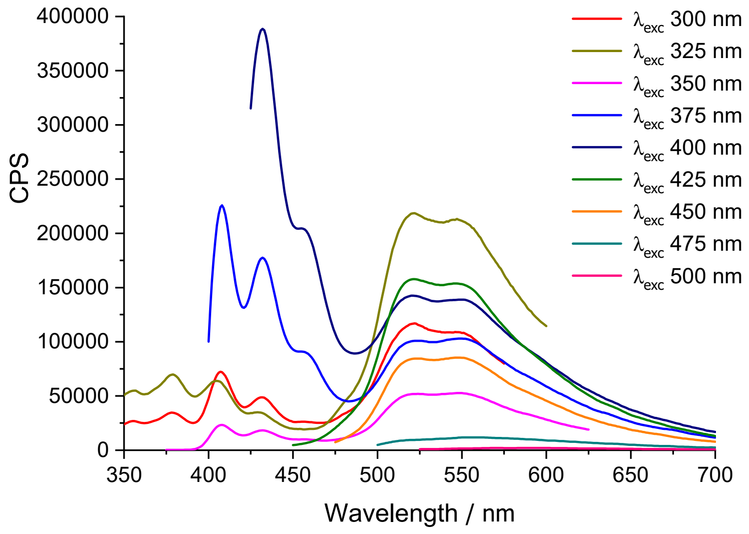

Figure S18. Emission-spectra of **4** measured in DMSO solution.

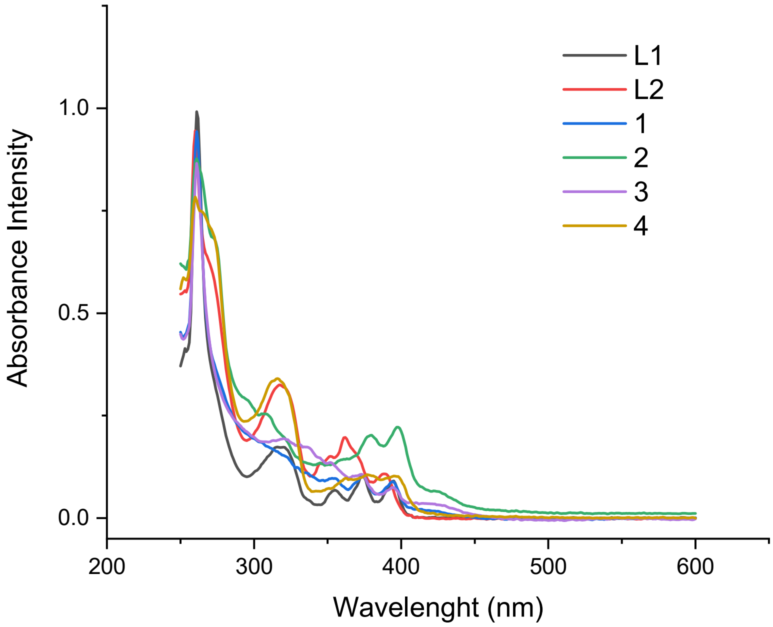

Figure S19. Absorption spectra in DMSO solution. **L1** and **L2** at 2·10^{-5}M and complexes **1-4** at 10^{-5}M.

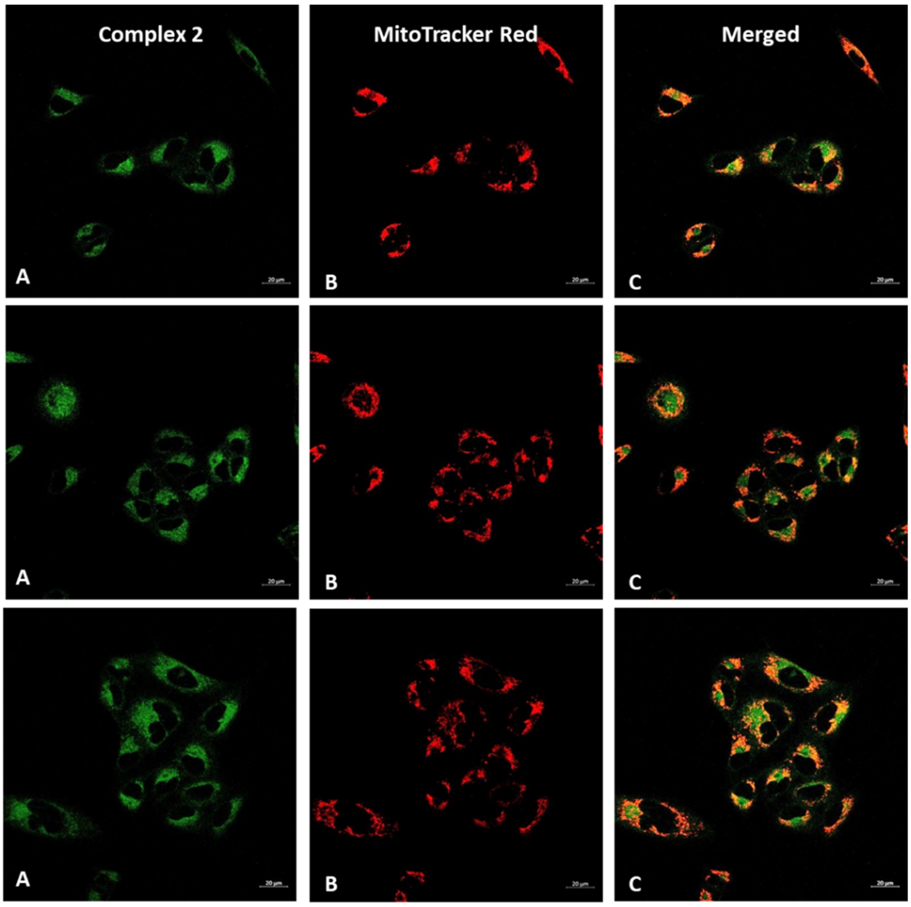

Figure S20. Fluorescence confocal microscopy images of A549 cells incubated with **2** (2h) and stained with MTR. (A) Image after irradiation at 458 nm; (B) after irradiation at 588 nm; (C) superimposition picture of A, B; Green: complex **2**, Red: MTR.

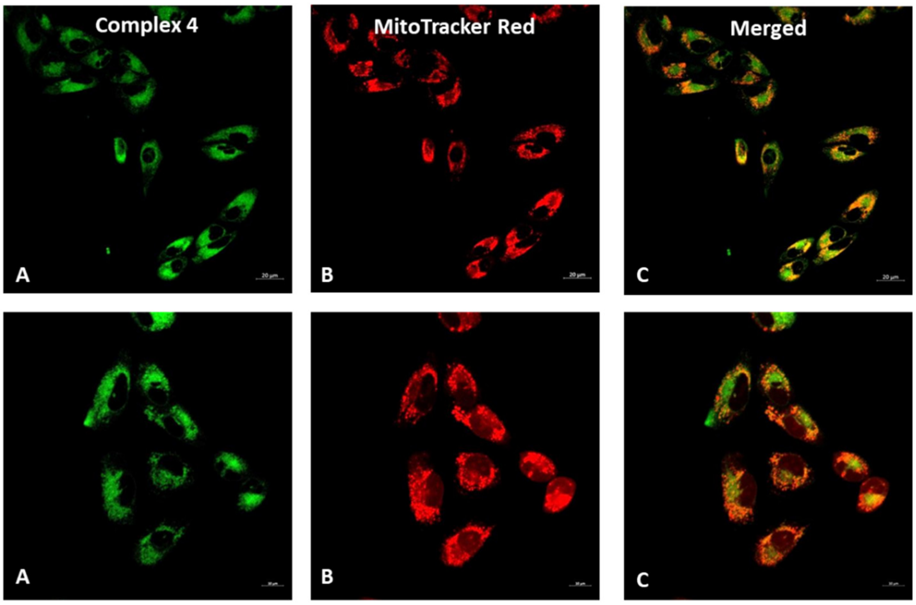

Figure S21. Fluorescence confocal microscopy images of A549 cells incubated with **3** (2h) and stained with MTR. (A) Image after irradiation at 458 nm; (B) after irradiation at 588 nm; (C) superimposition picture of A, B; Green: complex **3**, Red: MTR.

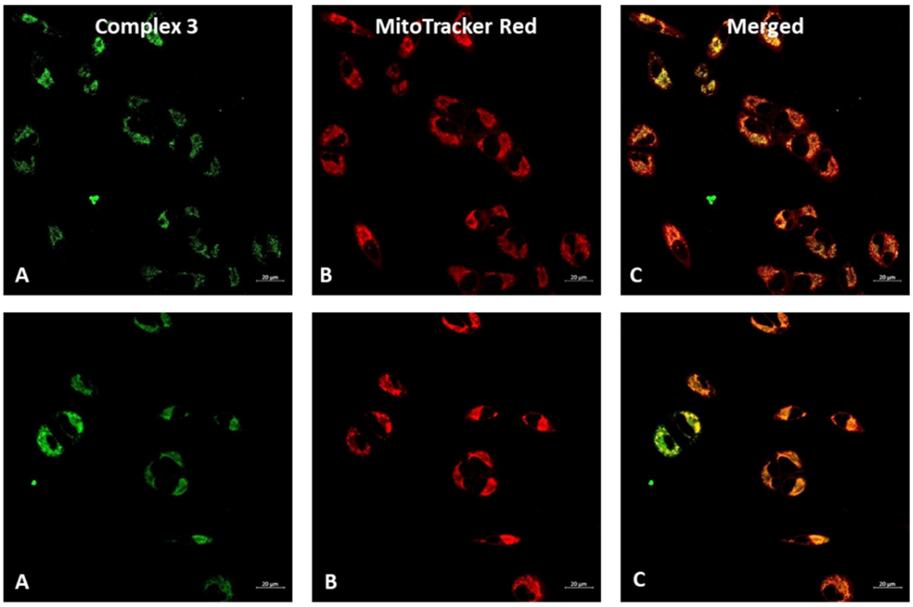

Figure S22. Fluorescence confocal microscopy images of A549 cells incubated with **4** (2h) and stained with MTR. (A) Image after irradiation at 458 nm; (B) after irradiation at 588 nm; (C) superimposition picture of A, B; Green: complex **4**, Red: MTR.
